# Mediating pathways between attention deficit hyperactivity disorder and type 2 diabetes mellitus: evidence from a two-step and multivariable Mendelian randomization study

**DOI:** 10.1017/S2045796024000593

**Published:** 2024-10-28

**Authors:** J. Zhang, Z. K. Chen, R. D. Triatin, H. Snieder, C. H. L. Thio, C. A. Hartman

**Affiliations:** 1 Department of Epidemiology, Unit of Genetic Epidemiology and Bioinformatics, University Medical Center Groningen, University of Groningen, Groningen, The Netherlands; 2 Division of Communicable Disease Control and Prevention, Shenzhen Center for Disease Control and Prevention, Shenzhen, Guangdong, China; 3 Faculty of Medicine, Department of Biomedical Sciences, Universitas Padjadjaran, Bandung, Indonesia; 4 Department of Population Health Sciences, Institute for Risk Assessment Sciences, University of Utrecht, Utrecht, The Netherlands; 5 Interdisciplinary Centre Psychopathology and Emotion Regulation, University Medical Center Groningen, University of Groningen, Groningen, The Netherlands

**Keywords:** attention deficit hyperactivity disorder, chronic conditions, health outcomes, metabolism

## Abstract

**Aims:**

Type 2 diabetes (T2D) is a global health burden, more prevalent among individuals with attention deficit hyperactivity disorder (ADHD) compared to the general population. To extend the knowledge base on how ADHD links to T2D, this study aimed to estimate causal effects of ADHD on T2D and to explore mediating pathways.

**Methods:**

We applied a two-step, two-sample Mendelian randomization (MR) design, using single nucleotide polymorphisms to genetically predict ADHD and a range of potential mediators. First, a wide range of univariable MR methods was used to investigate associations between genetically predicted ADHD and T2D, and between ADHD and the purported mediators: body mass index (BMI), childhood obesity, childhood BMI, sedentary behaviour (daily hours of TV watching), blood pressure (systolic blood pressure, diastolic blood pressure), C-reactive protein and educational attainment (EA). A mixture-of-experts method was then applied to select the MR method most likely to return a reliable estimate. We used estimates derived from multivariable MR to estimate indirect effects of ADHD on T2D through mediators.

**Results:**

Genetically predicted ADHD liability associated with 10% higher odds of T2D (OR: 1.10; 95% CI: 1.02, 1.18). From nine purported mediators studied, three showed significant individual mediation effects: EA (39.44% mediation; 95% CI: 29.00%, 49.73%), BMI (44.23% mediation; 95% CI: 34.34%, 52.03%) and TV watching (44.10% mediation; 95% CI: 30.76%, 57.80%). The combination of BMI and EA explained the largest mediating effect (53.31%, 95% CI: −1.99%, 110.38%) of the ADHD–T2D association.

**Conclusions:**

These findings suggest a potentially causal, positive relationship between ADHD liability and T2D, with mediation through higher BMI, more TV watching and lower EA. Intervention on these factors may thus have beneficial effects on T2D risk in individuals with ADHD.

## Introduction

Attention deficit/hyperactivity disorder (ADHD) is one of the most prevalent childhood psychiatric disorders affecting around 5–7% of children (Faraone *et al.*, 2015; Polanczyk *et al.*, 2014; Thomas *et al.*, 2015). It is characterized by extensive hyperactive, impulsive and inattentive behaviours that impair daily functioning, e.g. at school, work or in social relations (Breslau *et al.*, 2011; Fleming *et al.*, 2017; Ros and Graziano, 2018). In most children, ADHD persists during adolescence and into adulthood, either at full syndromal or subthreshold clinical levels (Faraone *et al.*, 2006). Prevalence estimates of ADHD in adulthood are 2–3% (Simon *et al.*, 2009).

Type 2 diabetes (T2D) is a multifactorial disorder in which impaired insulin secretion and/or insulin resistance results in dysregulated carbohydrate, lipid and protein metabolism (DeFronzo *et al.*, 2015). T2D is typically an adult-onset disease manifesting at middle or older ages (Carstensen *et al.*, 2020; Sun *et al.*, 2022), although more recently a substantial increase among younger people (aged <40 years) is observed, significantly boosting premature morbidity and mortality (Magliano *et al.*, 2020; Viner *et al.*, 2017). The global prevalence has been continuously rising over the past few decades, with T2D projected to affect 12.2% (783 million) of the world population by the year 2045 (Sun *et al.*, 2022), thus posing an increasingly unsustainable global health burden (Vos *et al.*, 2020).

Epidemiological studies have shown a higher T2D prevalence (up to 70%) among individuals with ADHD compared to the general population (Chen *et al.*, 2018b, 2018a). Meta-analysis estimated a twofold higher risk of T2D in individuals with ADHD (Garcia-Argibay *et al.*, 2023). In addition, recent evidence suggests an earlier onset of T2D in those with ADHD than those without (Chen *et al.*, 2018a). As a childhood onset condition, ADHD manifests much earlier than T2D, suggesting that ADHD, or factors/behaviours related to ADHD, precede and possibly cause T2D. However, it is currently unclear if the association between ADHD and T2D indeed represents a causal link and, if such link exists, how ADHD could lead to the onset of T2D.

Several factors could explain a potential link between ADHD and T2D. First, known precursors of T2D, such as obesity (Cortese *et al.*, 2016; Güngör *et al.*, 2016) and sedentary behaviour (Cook *et al.*, 2015), are also more common in individuals with ADHD compared to those in the general population. It is plausible that the behaviours involved in ADHD such as impulsivity may enhance the chance of overeating or poor diet, leading to obesity (Cortese and Castellanos, 2014) in turn leading to T2D (Landau and Pinhas-Hamiel, 2019). Also, screen time utilization is longer among individuals with ADHD and may partly explain the relation between sedentary behaviours and T2D (Nightingale *et al.*, 2017; Yang *et al.*, 2022). Second, ADHD is strongly linked to lower educational attainment (EA) (Fleming *et al.*, 2017; Korrel *et al.*, 2017), potentially due to early school dropout or poor school performance, although such causal pathways are currently unclear (Hartman, 2020). Evidence from observational studies and genetic studies have suggested that lower EA and T2D are causally linked (Agardh *et al.*, 2011; Zhang *et al.*, 2022). Lower EA may thus be an important pathway connecting ADHD and T2D. Third, individuals with ADHD are at increased risk for cardiovascular diseases (Akmatov *et al.*, 2021; Chen *et al.*, 2018b; Li *et al.*, 2022b, 2023), elevated blood pressure (Chen *et al.*, 2018b) and increased peripheral inflammation (Saccaro *et al.*, 2021). These well-known risk factors of T2D (Emdin *et al.*, 2015; Wang *et al.*, 2012) may also be mediators between ADHD and T2D, although a recent register-based study focused on referred and diagnosed patients (i.e. more severely affected patients) suggests that cardiovascular traits played only a minor mediating role (Garcia-Argibay *et al.*, 2023). Despite the plausibility of these pathways, it is not known whether, and to what extent, these are mechanisms explaining the association of ADHD with T2D. A better understanding of causal mechanisms may help prevention of T2D in individuals with ADHD. Therefore, research on mediating pathways is needed (Byrne *et al.*, 2017; Hartman, 2020).

A widely applied method that supports causal inference from observational data is Mendelian randomization (MR), which uses genetic instrumental variables to examine the relationship between a risk factor (in this case ADHD), and a disease outcome (T2D) (Smith and Ebrahim, 2003). Under a number of assumptions, MR yields a causal estimate, i.e. an estimate that is less likely to be biased due to confounding, the primary source of bias in observational studies (Smith and Ebrahim, 2004). According to Mendel’s laws of random segregation and independent assortment, alleles are assigned randomly before conception, independently of other traits. Thus, genetic variants can be exploited as a natural experiment. Recent advances in MR methodology include multivariable MR (MVMR), which among other things can be applied to investigate mediation (Sanderson, 2021).

In recent years, MR has been applied to study effects of ADHD on a wide range of outcomes (*see, for an overview,* Riglin and Stergiakouli, 2022), including but not limited to ischemic stroke (Du *et al.*, 2023), Parkinson’s disease (Li *et al.*, 2020), insomnia (Gao *et al.*, 2019), autism spectrum disorder (Baranova *et al.*, 2022), body mass index (BMI) or obesity (Karhunen *et al.*, 2021; Liu *et al.*, 2021; Martins-Silva *et al.*, 2019), substance use (Treur *et al.*, 2021) and socio-economic status (SES) (Michaëlsson *et al.*, 2022). One previous MR study reported a positive relationship between ADHD and T2D (Leppert *et al.*, 2020). Our study aimed to improve on previous studies in two ways. First, we aimed to update ADHD-T2D effect estimates using the most recent genome-wide association studies (GWAS) on ADHD (Demontis *et al.*, 2023) and T2D (Mahajan *et al.*, 2018), which, due to their larger sample sizes, have yielded more precise estimates of genetic effects. Second, we aimed to identify potential mediating pathways that link ADHD to T2D, specifically BMI, sedentary behaviour, EA, smoking, C-reactive protein (CRP), systolic blood pressure (SBP), diastolic blood pressure (DBP).

## Methods

### Study design

This is an MR study that investigated the relation between ADHD and T2D, and potential mediation through BMI, childhood obesity, childhood BMI, sedentary behaviour (daily hours of TV watching), blood pressure (SBP, DBP), CRP and EA. MR uses genetic instruments to genetically predict an exposure trait. Here, we used single nucleotide polymorphisms (SNPs) as genetic instruments. MR yields causal estimates under three key assumptions: (1) relevance, (2) exchangeability and (3) exclusion restriction (for more details, see **ESM Methods**). We used two-sample MR (2SMR) methods that uses SNP-trait associations available from GWAS summary data (Burgess *et al.*, 2013). We obtained summary statistics of the genetic associations from the most recent GWAS for each respective phenotype (details in Table 1). To determine whether a trait mediates the effect between exposure and outcome, two-step 2SMR was performed (Relton and Davey Smith, 2012). The first step involves genetically predict ADHD and estimating its association with potential mediators. The second step involved genetically predicting these mediators and estimating their effect on the outcome while accounting for ADHD using MVMR. Then, the overall effect of ADHD was separated into an indirect effect (i.e. the effect of ADHD on T2D via the mediator) and a direct effect (i.e. the effect of ADHD on T2D independent of the mediator). The distinct analysis steps for mediation analysis, as well as the decision algorithm on which variables to take forward to the subsequent step, are outlined in Fig. 2. Reporting of the present study was done in accordance with STROBE-MR guidelines (**ESM STROBE-MR**) (Skrivankova *et al.*, 2021a, 2021b).

**Table 1.** Overview of GWAS data used

Phenotype	Unit	Number of participants	Number of lead SNPs	Explained variance by lead SNPs	Ancestry	Consortium/ cohort	Author	Year of publication	PubMed ID
ADHD	Log-odds	38,691 cases 186,843 control participants	27	\	European	iPSYCH + deCODE + PGC	Demonis et al.	2023	36702997
Childhood BMI	kg/m^2^	61,111	25	3.6%	European	ECG	Vogelezang et al.	2020	33045005
Childhood obesity	Yes vs no	13,005 cases 15,599 control participants	18	\	European	ECG	Bradfield et al.	2019	31504550
Years of schooling	SD (4.2 years)	1,131,881	1271	11%	European	SSGAC	Lee et al.	2018	30038396
BMI	kg/m^2^	681,275	941	6%	European	GIANT	Yengo et al.	2018	30124842
SBP	mmHg	775,601	970	5.7%	European	UKBB + ICBP	Evangelou et al.	2018	30224653
DBP	mmHg	775,601	962	5.3%	European	UKBB + ICBP	Evangelou et al.	2018	30224653
Type 2 diabetes	Yes vs no	74,124 cases 824,006 control participants	403	18%	European	DIAGRAM	Mahajan et al.	2018	30297969
Smoking initiation	Ever vs never	557,337 cases 674,754 control participants	378	2.3%	European	GSCAN	Liu et al.	2019	30643251
CRP	mg/L	204,402	58	7%	European	CIWG	Ligthart et al.	2018	30388399
TV watching	SD (1.5 hours)	408,815	152	2.3%	European	UKBB	van de Vegte et al.	2020	32317632

PGC, Psychiatric Genomics Consortium; DIAGRAM, DIAbetes Genetics Replication And Meta-analysis; EGG, Early Growth Genetics; GIANT, Genetic Investigation of Anthropometric Traits; GSCAN, GWAS & Sequencing Consortium of Alcohol and Nicotine use; ICBP, International Consortium for Blood Pressure; SSGAC, Social Science Genetic Association Consortium; UKBB, UK Biobank; CIWG, Cohorts for Heart and Aging Research in Genomic Epidemiology (CHARGE) Inflammation Working Group.

### Variable definitions

The original GWAS defined T2D using a diagnostic fasting glucose, casual glucose level or plasma glucose level of 2 hours or an HbA_1c_ level; use of glucose-lowering medication (by Anatomical Therapeutic Chemical code or self-report); or a history of T2D based on electronic medical records, self-report or a combination of these (Mahajan *et al.*, 2018).

In the original GWAS (Demontis *et al.*, 2023), ADHD cases were diagnosed by psychiatrists at in- or out-patient clinics according to the ICD10 criteria (F90.0, F90.1, F98.8 diagnosis codes) or individuals that have been prescribed medication specific for ADHD symptoms (ATC-NA06BA, mostly methylphenidate).

In selecting mediators, we considered potential for modification, observational epidemiological evidence that link these to both ADHD or T2D, as well as the availability of comprehensive GWAS data. BMI was calculated by dividing weight (kg) by height squared (m^2^) (Yengo *et al.*, 2018). Sedentary behaviour was proxied by daily hours of TV watching (in standard deviations, 1.5 hours) (van de Vegte *et al.*, 2020). SBP and DBP were derived from two automated or two manual blood pressure measurements (Evangelou *et al.*, 2018). Smoking was defined as ever-smoking vs never-smoking (Liu *et al.*, 2019). EA was defined as years of schooling (in standard deviations, 4.2 years) based on the International Standard Classification of Education (ISCED) 2011 (Lee *et al.*, 2018; UNESCO Institute for Statistics., 2012). Serum CRP was measured in mg/L using standard laboratory techniques, and was transformed by its natural logarithm in the original GWAS (Ligthart *et al.*, 2018). Childhood obesity was defined as ≥95th percentile of BMI achieved 2–18 years old (Bradfield *et al.*, 2019), and childhood BMI was measured in children aged between 2 and 10 years (Vogelezang *et al.*, 2020).

### Instrument selection

**Figure 1.**
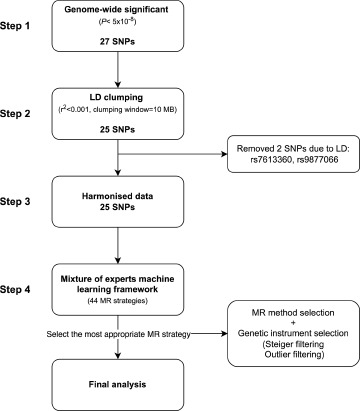
ADHD instrument selection for ADHD-T2D association. LD, linkage disequilibrium; T2D, type 2 diabetes; MR, Mendelian randomization.

In Table 1, all identified SNPs, and their associations with T2D, mediators, and ADHD were extracted from summary GWAS data. From the most recent GWAS meta-analysis of ADHD, which included 38,691 people with ADHD and 186,843 controls, 27 SNPs (*p* < 5 × 10^−8^) were chosen as genetic instruments for ADHD (Demontis *et al.*, 2023). We applied strict linkage disequilibrium (LD) clumping thresholds for ADHD genetic instruments (LD cut-off of *r*^2^ < 0.001 within a window of 10 MB), leading to the removal of 2 out of 27 SNPs. We oriented SNP alleles for ADHD towards positive coefficients, and harmonized the SNP alleles from the outcome GWAS accordingly. We inferred the strand for palindromic SNPs using allele frequencies, and removed ambiguous palindromes (minor allele frequency between 0.42 and 0.58). Figure 1 shows the SNP selection procedure for the ADHD-T2D analysis. It should be noted that the ADHD genetic effect was estimated on the liability scale and not on the yes/no scale. We applied the same criteria to select genetic instruments for each mediator (see Table 1 for details on each GWAS). Detailed information on SNPs and their associations with ADHD, mediators and T2D can be found in **ESM SNP Data**.

### Univariable MR analysis

To estimate the univariable associations between genetically predicted ADHD and T2D, and between genetically predicted ADHD and mediators we used a number of methods. First, we performed conventional random-effects inverse variance weighted (IVW) MR of single-SNP Wald ratios (SNP-outcome divided by SNP-exposure association), examining heterogeneity statistics to assess potential pleiotropy, and Egger intercepts to assess potential directional pleiotropy. In addition, we performed a large range of MR sensitivity analyses. In total, we used 44 univariable MR strategies (11 distinct MR estimation methods × Steiger filtering yes/no × outlier filtering yes/no). We applied the MR mixture-of-experts (MR-MoE) machine learning framework to assist in selecting the MR estimate most likely to be reliable (Hemani *et al.*, 2017). MR-MoE prioritizes methods based on certain characteristics of the data such as heterogeneity and directional pleiotropy, and discards instruments that are possibly invalid (e.g. potentially pleiotropic outliers and/or ‘reverse causal’ SNPs). The top ranked MR estimates for each univariable association were taken forward to further analysis.

### MVMR analysis

We estimated the ADHD-adjusted association between each mediator and T2D risk using regression-based MVMR-IVW (Burgess and Thompson, 2015), using trait-specific instruments in addition to ADHD instruments.

### Mediation analysis

To calculate the indirect effect of each individual mediator (childhood BMI/obesity, BMI, SBP, DBP, smoking, CRP, EA and TV watching), we used the product-of-coefficients approach. This involved multiplying the ADHD–mediator association (derived from univariable MR) with the ADHD-adjusted association between mediator and outcome (Burgess *et al.*, 2015). The indirect effect was divided by the total effect to assess the proportion of the overall effect of ADHD on T2D that was mediated by each individual mediator. We used the bootstrap method and delta method to estimate the confidence interval for the indirect effect and the proportion mediated.

We investigated indirect effects of multiple mediators combined (e.g. BMI + EA) using the difference in regression coefficient method. This involved subtracting the direct effect of ADHD on T2D (after adjustment for the mediators in MVMR) from the total effect of ADHD on T2D (from univariable MR), to obtain the indirect effect through multiple mediators. To identify the combination with the largest proportion mediated, and to evaluate potential overlapping effects between mediators, we looked into all combinations of mediators.

**Figure 2.**
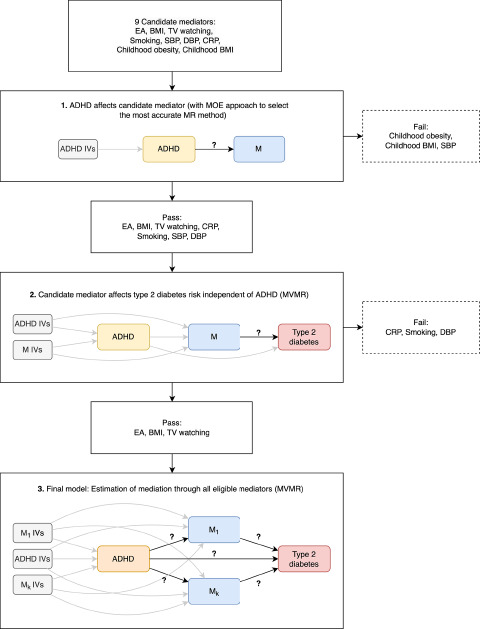
Decision algorithm for mediator selection in the final analysis. MR, Mendelian randomization; MVMR, Multivariable Mendelian randomization; MoE, mixture of experts; ADHD, Attention deficit hyperactivity disorder; EA, educational attainment; BMI, body mass index; TV watching, television watching; SBP, systolic blood pressure; DBP, diastolic blood pressure; CRP, C-Reactive Protein; IV, instrument variable.

Mediators were selected into the final analysis if they met the following requirements: (1) ADHD affects the mediator in univariable MR; (2) The mediator affects T2D risk independent of ADHD in an MVMR model (see Fig. 2).

### Sensitivity analyses

As an alternative to the product-of-coefficients method to calculate indirect effects through individual mediators, we used the difference in regression coefficient method. The robustness of the MVMR-IVW results was evaluated using the MVMR-Egger method (Rees *et al.*, 2017). We investigated potential bidirectional relationship between ADHD and possible mediators in reverse MR analysis (genetic instruments for each mediator as the exposure).

All MR analyses were conducted using R (version 4.2.1) (Team RC, 2014) and the *TwoSampleMR* R package v0.5.7 (Hemani *et al.*, 2018).

## Results

### Univariable MR analysis

We included 25 SNPs as genetic instruments for ADHD (Fig. 1). Conventional random-effects IVW estimated a significant positive association between ADHD liability and T2D (OR: 1.15; 95% CI: 1.12, 1.18) in the presence of heterogeneity (*Q* [*df*] = 102.2 [24], heterogeneity *p*-value = 1.26 × 10^−11^) butabsence of directional pleiotropy (Egger intercept = −1.50 ± 1.59, Egger intercept *p*-value = 0.357) (**ESM Table S1**). In the results of all 44 univariable MR strategies, associations of ADHD liability with T2D ranged from OR 0.99 (simple mean, no Steiger filtering, no outlier filtering) to OR 1.28 (random effects MR Egger, Steiger filtering, no outlier filtering). Overall, results from the various MR strategies converged to a positive association. MoE assigned the weighted median MR estimate (no Steiger filtering, no outlier filtering) to be the most reliable, which estimated ADHD liability to associate with 10% higher odds of T2D (OR: 1.10; 95% CI: 1.02, 1.18; MoE score: 0.72, **ESM Table S2**).

**Figure 3.**
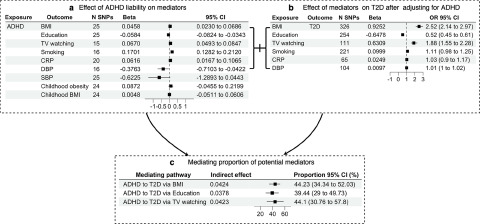
(a) MR-estimated effects of ADHD liability on each mediator separately, presented as Beta with 95% CI. (b) MR-estimated effects of each mediator separately on type 2 diabetes after MVMR adjustment for ADHD, presented as Beta /OR with 95% CI. (c) MR-estimated effects of indirect effects of each mediator separately, by product-of-coefficients method with bootstrap method-estimated 95% CIs. MR-estimated proportions mediated (%) are presented with 95% CIs. OR, odds ratio; CI, confidence interval; ADHD, Attention deficit hyperactivity disorder; BMI, body mass index; TV watching, television watching; SBP, systolic blood pressure; DBP, diastolic blood pressure; CRP, C-Reactive Protein; T2D, type 2 diabetes.

Associations of genetically predicted ADHD liability with each candidate mediator are shown in Fig. 3a. In the MR models prioritized by MoE (**ESM Table S2**), genetically predicted ADHD liability associated with higher BMI (*β* = 0.05 kg/m^2^; 95% CI: 0.02, 0.07); more TV watching (*β* = 0.07 SD of TV watching; 95% CI: 0.05, 0.08, translating to 0.10 h more TV watching); higher odds of smoking (OR: 1.19; 95% CI: 1.14, 1.24); higher level of circulating CRP (*β* = 0.06; 95% CI: 0.02, 0.11), lower EA (*β* = −0.06 SD in years of schooling; 95% CI: −0.08, −0.03, translating to 0.25 less years of schooling). Genetically predicted ADHD liability was not associated with childhood obesity or childhood BMI. We observed an inverse association between liability of ADHD and blood pressure (SBP: *β* = −0.62; 95% CI: −1.29, 0.04; DBP: *β* = −0.38; 95% CI: −0.71, −0.04). Six potential mediators were taken forward to the next step, i.e. MVMR analysis of the ADHD-adjusted effect of mediators (i.e. BMI, TV watching, smoking, CRP, EA and DBP) on T2D (Fig. 2).

### MVMR analysis

Figure 3b shows associations of genetically predicted mediators on T2D with adjustment for ADHD using MVMR. A 1 kg/m^2^ higher genetically predicted BMI associated with 2.52 times higher odds of T2D (95% CI: 2.14, 2.97). One SD (4.2 years of schooling) higher genetically predicted EA associated with lower odds of T2D (OR: 0.52; 95% CI: 0.45, 0.61). One SD (1.5 hours) of genetically predicted TV watching associated with 1.88 times higher odds of T2D (95% CI: 1.55, 2.28). No associations with T2D were found for genetically predicted smoking (OR: 1.11; 95% CI: 0.98, 1.25), CRP (OR: 1.03; 95% CI: 0.90, 1.17) and DBP (OR: 1.01; 95% CI: 0.99, 1.02). Consequently, BMI, EA and TV watching were taken forward to the final mediation analysis.

### Mediation analysis

Figure 3c displays the proportion of the effect of ADHD liability on T2D explained by each mediator separately. BMI mediated 44.23% (95% CI: 34.34%, 52.03%) of the total effect of ADHD liability on T2D. EA mediated 39.44% (95% CI: 29.00%, 49.73%) of the total effect, whereas TV watching mediated 44.10% (95% CI: 30.76%, 57.80%).

We examined the proportion mediated of different combinations of BMI, TV watching and EA. This was done in an effort to find the combination that explained the most variance in the ADHD–T2D association, as well as to investigate potential overlap in effects between mediators.

Combining EA with any one of the other mediators resulted in a combined proportion mediation estimate of 40–50% (Table 2). Among the two-mediator combinations, EA + BMI explained the largest combined mediation effect (53.31; 95% CI: −1.99%, 110.38%) of the estimated effect of ADHD liability on T2D. The EA + TV combination showed subtly lower proportion mediated (51.28%; 95% CI: −20.90%, 123.89%). BMI + TV showed less mediating effect (26.87%, 95% CI: −36.96%, 90.89%), suggesting overlapping mediating pathways between higher BMI and TV watching.

**Table 2.** Estimates of proportion mediated by combinations of factors

			IDE and 95% CI			Prop and 95% CI		
Combinations	Total effect	Direct effect	IDE	LL	UL	Prop	LL	UL
BMI + EA	0.0958	0.0448	0.0511	−0.0019	0.1058	53.31	−1.99	110.38
BMI + TV	0.0958	0.0701	0.0258	−0.0350	0.0871	26.87	−36.96	90.89
EA + TV	0.0958	0.0467	0.0492	−0.0200	0.1188	51.28	−20.90	123.89
BMI + EA + TV	0.0958	0.0518	0.0441	−0.0065	0.0946	45.99	−6.80	98.73

IDE, indirect effect; LL, lower limit; UL, upper limit; CI, confidence interval; Prop, proportion; BMI, body mass index; EA, educational attainment; TV, television.

The full three-mediator combination (BMI + EA + TV) did not result in a higher estimate of combined proportion mediated (45.99%, 95% CI: −6.80%, 98.73%), again suggesting overlapping effects between these three. For all mediator combinations, delta method estimation of confidence intervals was consistent with the bootstrap method, with wider intervals that all included zero (**ESM Table S3**).

### Sensitivity analyses

To assess the consistency of our main MR product-of-coefficients estimates of individual mediation, we performed additional MVMR mediation analysis using the difference in regression coefficient method. Although the estimated indirect effect through BMI was lower, estimates for EA and TV watching were generally similar (**ESM Figure S1**). Results from MVMR-Egger sensitivity analyses showed no significant effects (**ESM Table S4**). However, given the absence of evidence for directional pleiotropy, we consider the MVMR-IVW estimates to be reliable. There was reasonable instrument strength (*F* > 10) of SNPs for EA, BMI and TV in all MVMR analyses. However, conditional instrument strength for ADHD was low.

In reverse MR analyses, higher EA suggestively reduced liability of ADHD (OR: 0.30, 95% CI: 0.26, 0.35), whereas more smoking, hours of TV watching and a higher BMI increased the liability of ADHD (**ESM Table S5, ESM Figure S2**).

## Discussion

This study used a two-step MVMR approach to test a putative causal effect of ADHD on T2D, and to explore potential mediators in this relation. ADHD, instrumented by 25 SNPs, was associated with 10% higher odds of T2D (OR: 1.10). Individually, BMI, EA and TV watching mediated 39–44% of the relation, with up to 53% mediation when combining multiple mediators. However, confidence intervals were wide and included zero for each combination. While a simulation study has shown little evidence of bias in MR point estimates of mediation effect, the indirect effect and the proportion mediated estimate may have large error terms in case of a modest total effect (Carter *et al.*, 2021). Therefore, the estimated proportions mediated are likely reliable, but some caution in interpretation is warranted for the error terms.

For the effect magnitude of ADHD on T2D, our study corroborated the estimate of a previous MR study that used 11 ADHD SNPs (OR: 1.09) (Leppert *et al.*, 2020). This estimate is however smaller than the OR reported in a recent study that used 26 instruments (OR: 1.30) (Baranova *et al.*, 2023). Using the same methods and GWAS data as described in the Baranova study, we however reassuringly arrived at an effect estimate similar to our main analysis (OR: 1.10, **ESM Table S6, ESM SNP data 9**), thus we were unable to replicate the larger Baranova estimate of OR 1.30. Results from a meta-analysis and an observational study also demonstrated a positive association (OR: 2.29 and HR: 2.35, respectively) between ADHD and T2D (Garcia-Argibay *et al.*, 2023). It must be mentioned that MR results are necessarily on the liability scale (i.e. per log-odds unit increase in genetic liability to ADHD) rather than the binary scale (ADHD yes/no), thus the observational estimate cannot be directly compared to our MR estimates.

We found evidence that BMI mediates the effect of ADHD on T2D. Multiple studies have demonstrated that ADHD liability is associated with an increased risk of obesity (Karhunen *et al.*, 2021; Nigg *et al.*, 2016) or higher BMI (Liu *et al.*, 2021). Garcia-Argibay *et al.* (2023) also found observational evidence of a mediating role of obesity. We extended the evidence that this mediating pathway is causal. Impaired inhibitory control and reward sensitivity that characterize ADHD could result in unhealthy, irregular eating habits (Cortese and Castellanos, 2014) thus leading to obesity. Given that obesity, or elevated BMI are thought to be the primary causes of T2D (Censin *et al.*, 2019; Sun *et al.*, 2022), the indirect pathway from ADHD to obesity/higher BMI to T2D might be the most relevant.

Furthermore, we found that part of the association between ADHD and T2D is driven by lower educational attainment, which is related to lower SES, and accordingly lower financial security, lower quality employment and less job security (Galobardes *et al.*, 2006a, 2006b). These SES-related risk factors are associated with T2D, potentially due to poorer nutrition, poorer health behaviours, less healthcare seeking and poorer access to (high quality) healthcare (Gavin *et al.*, 2024; Krishnan *et al.*, 2010; Zhang *et al.*, 2022). Therefore, associations between ADHD and T2D may not only be driven by educational attainment, but also partly be driven by the socio-economic context into which individuals with ADHD are sorted by the educational system (Schmengler *et al.*, 2023). Thus, ADHD related symptoms, such as forgetfulness, and difficulty in engaging and sustaining attention, could induce poor performance in school (Jangmo *et al.*, 2019). As a consequence, children with ADHD are more often assigned to lower educational tracks in selective educational systems, subsequently leading to lower educational attainment, illustrating the health-related selection into lower SES (Schmengler *et al.*, 2023).

Next to BMI and EA, our study suggests sedentary behaviour, measured by TV watching, is a mediator. Those with ADHD are observed to engage in more screen time (Ansari and Crosnoe, 2016), with dose-dependency on severity of ADHD (Montagni *et al.*, 2016; Vaidyanathan *et al.*, 2021). Possibly, ADHD people are prompted to watch TV or gaming to seek arousal, or to avoid social difficulties (Roberti, 2004; Vandewater *et al.*, 2005). Such sedentary behaviour could lead to increases in trunk and body fat percentage, thereby increasing risk of T2D (Li *et al.*, 2022a).

The mediation effects of the three pathways described above are not expected to be independent of each other, given the strong (genetic) correlations and potential causal relationships between BMI, EA and sedentary behaviour (Cassidy *et al.*, 2017; van de Vegte *et al.*, 2020; Zhang *et al.*, 2022). We therefore performed analyses in which we modelled combined mediation effects. These indeed yielded non-additive results, i.e. combined effects through all three mediators were smaller than the sum of the individual effects, corroborating overlap in mediation effects. Our MVMR results show that each individual mediator generally retains significant effects on T2D conditional on the other mediators, suggesting partially independent effects and thus incomplete overlap. We additionally found there that nearly half of the total effect remains unexplained by BMI, EA and TV watching. Other mediating pathways thus likely exist. A recent register-based study suggests the observed association between ADHD and T2D is largely explained by psychiatric comorbidities, with unhealthy behaviours (smoking and drinking), dietary habits and neurobiological abnormalities proposed as possible explanations (Garcia-Argibay *et al.*, 2023). Once sufficiently large GWAS studies on these potential additional mediators are available, it would be possible to assess their indirect effects through MR.

The difference of coefficients method returned a much lower estimate of proportion mediated by BMI than our main products method (16% vs 44%). Possibly, this is due to non-collapsibility of the odds ratio. The mediation literature recommends the product-of-coefficients method, but binary outcomes must have a low prevalence (i.e. <10%), so that the odds ratio approximates the linear risk ratio (Vanderweele and Vansteelandt, 2010). In case of common outcomes (i.e. prevalence >10%), estimates from the product-of-coefficients method and difference of coefficients method are unlikely to perfectly align (Carter *et al.*, 2021). In the present study, both methods agreed on mediation by BMI, EA and TV watching. Nevertheless, some caution is warranted with regard to the differing BMI estimates, as well as our estimates of combined proportion mediated, for which we necessarily used the difference method.

In sensitivity reverse MR analysis, we found surprising results suggestive of reverse causation, i.e. several of our candidate mediators (i.e. smoking, BMI, EA, TV watching) are suggested to cause ADHD. Such effects of smoking and EA on ADHD were also reported in another MR study (Soler Artigas *et al.*, 2023). There is evidence that physical exercise mitigates ADHD symptoms (Choi *et al.*, 2015; Rommel *et al.*, 2013) and some evidence that screen time reduces executive functioning (Liu *et al.*, 2022) and thus, some bidirectionality is possible. However, we find it highly unlikely that these factors cause onset of ADHD. A plausible explanation is that MR estimates (both in forward and reverse analyses) are affected by biased GWAS estimates induced by (spurious) gene-environment correlation (Quinn and D’Onofrio, 2020). Also, gene-trait associations are possibly mediated through the family environment due to assortative mating (partner choice based on similarity, resulting in non-random distribution of genetic variants) (Howe *et al.*, 2019), dynastic effects (effects of non-transmitted alleles that affect traits through the environment) (Kong *et al.*, 2018). Alternatively, reverse causation could be due to inherited alleles, i.e. parental EA and smoking, for instance, would affect offspring ADHD risk and severity of symptoms. Genetic propensity towards lower EA and smoking in individuals with ADHD could thus be inherited from the parents. Indeed, there is evidence that the above described phenomena could affect genetic studies into ADHD. One study found evidence that liability of earlier age at first sexual intercourse and of lower rate of past tobacco smoking in non-heavy smokers increasing the odds of ADHD (Soler Artigas *et al.*, 2023), similar to the chronologically implausible reverse effect of EA on ADHD in our study. These apparent reverse effects may be driven by dynastic effects, as literatures linked young parental age or maternal smoking with increased risk of ADHD in children (Huang *et al.*, 2018; Hvolgaard Mikkelsen *et al.*, 2017). Assortative mating is also likely in individuals with ADHD, with a sevenfold higher odds of ADHD in the partner (Nordsletten *et al.*, 2016). Studies comparing within-family with population-based GWAS estimates found that within-sibship estimates are smaller than population estimates, for educational attainment, cognitive ability, depressive symptoms and smoking, with a shrinkage in SNP effects that ranged from 19% (smoking) to 50% (depressive symptoms) (Howe *et al.*, 2022). Although the authors did not investigate ADHD, their results suggests that SNP-estimates of population-based GWAS on cognition and mental health could in part be confounded by demographic effects (population stratification, assortative mating) and indirect genetic effects. In contrast, one twin study investigated within- and between-family differences in ADHD polygenic score effects on ADHD symptoms found little difference (Selzam *et al.*, 2019). This suggests that for ADHD, confounding by demographic and indirect genetic effects is negligible. Given all the above, although there is currently limited evidence of bias in population-based GWAS on ADHD, we cannot exclude the possibility that ADHD SNP estimates are biased through these phenomena. Within-family analysis is thought to be largely robust against these effects (Brumpton *et al.*, 2020; Davies *et al.*, 2019). Therefore, sufficiently large within-family data (e.g. parent-offspring trios, between-sibling design), currently unavailable for ADHD, is needed to account for such potential sources of bias in GWASs and MR and further validate our findings.

Given the evidence for a causal effect of ADHD on T2D, it is to be expected that successful management of ADHD will reduce T2D risk either directly or through increasing EA, reducing BMI and promoting non-sedentary behaviour in individuals with ADHD. Based on large registry data, it has been shown that treatment with ADHD medication increases school grades as well as the probability of completing upper secondary education (Jangmo *et al.*, 2019), while discontinuation of ADHD medication was associated with a (small) decline in grades (Keilow *et al.*, 2018). Similarly, test scores were higher during periods on rather than off medication (Lu *et al.*, 2017). Collaborative school-home behavioural interventions may also benefit educational outcomes (Pfiffner *et al.*, 2013). With regard to reducing BMI, it is known that stimulant treatment reduces appetite, and thus, weight loss, in children with overweight or obesity and ADHD, stimulant treatment yields an additional benefit in terms of weight management (Fast *et al.*, 2021). Similarly, we already mentioned that physical exercise reduces ADHD symptoms (Choi *et al.*, 2015) with the added advantage that this may benefit weight lowering. Finally, it is implausible that stimulant treatment reduces sedentary behaviour and screen time, but behavioural interventions, although not specifically studied in individuals with ADHD, may provide effective treatment (Jones *et al.*, 2021). Finally, high BMI in individuals with ADHD could additionally be targeted through diet (e.g. replacing a ‘Western-style’ diet with a healthy diet) (Howard *et al.*, 2011; Millichap and Yee, 2012). It has also been reported that altering the metabolic profile via restriction and elimination diets will reduce both ADHD symptoms and BMI level (Pelsser *et al.*, 2011).

Previous MR studies that investigated mediation generally used IVW estimates, which relies on the strong assumption of absence of horizontal pleiotropy. Given that horizontal pleiotropy is likely to be pervasive (Hemani *et al.*, 2017), using IVW estimates might not always be appropriate. We therefore relied on the MR-MoE tool which uses a reproducible, pre-defined machine learning algorithm to identify the most appropriate models and thus most reliable univariable MR estimates that were then taken forward to mediation analysis. For the MVMR setting however, relatively few pleiotropy robust methods are available, and to our knowledge, no machine learning algorithm for prioritizing MVMR models exists. Thus, the MVMR-IVW estimates of direct effects we used for mediation analysis may be biased by pleiotropy, even though estimates from MVMR-Egger were largely consistent. Future study, simulation or otherwise, may investigate potential machine learning algorithms to prioritize MVMR models.

This is the first study that systematically explores mediating mechanisms in the relation between ADHD and T2D using MR methods. We used the most comprehensive large-scale GWAS data available to us, optimizing the power and precision of our study. Several limitations must be acknowledged. First, we already mentioned that gene-environment correlation, assortative mating and dynastic effects may bias GWASs and by that MR. This awaits GWASs that use within-family data. Second, MR may be biased by pleiotropic effects of SNPs, i.e. genetic variants influencing the outcome through other pathways than the exposure, which would be a violation of exclusion restriction criterion. Therefore, we compared estimates from a wide range of pleiotropy robust MR analyses, which generally and reassuringly showed consistency. Nevertheless, MR makes several assumptions that are strong and untestable, and thus MR results should be interpreted with caution. Third, the present study assumes the absence of exposure × mediator interaction, the investigation of which requires large-scale individual level data and is therefore not possible with summary level data. Four, conditional instrument strength for ADHD in MVMR was low (*F* < 10). Future, more powerful GWAS may identify stronger instruments for ADHD with which our MVMR results may be corroborated. Fifth, if any sample overlap existed between GWAS studies it may have biased MR estimates towards observational, possibly confounded, association estimates (Burgess *et al.*, 2016). These limitations illustrate that more evidence is needed to firmly establish causality, by triangulating with future observational and (quasi-)interventional studies.

We conclude that there is a possible causal relationship between liability of ADHD and T2D, in which ADHD liability causes higher T2D risk through higher BMI, more TV watching and lower EA. Intervention on these factors may have beneficial effects on reducing T2D risk in individuals with ADHD.

## Supporting information

Zhang et al. supplementary material 1 Zhang et al. supplementary material

Zhang et al. supplementary material 2 Zhang et al. supplementary material

Zhang et al. supplementary material 3 Zhang et al. supplementary material

Zhang et al. supplementary material 4 Zhang et al. supplementary material

## Data Availability

GWAS data are publicly available through the MRC IEU Open GWAS database (https://gwas.mrcieu.ac.uk/). The analysis code can be shared upon request.

## Supplementary material

The supplementary material for this article can be found at https://doi.org/10.1017/S2045796024000593.

## References

[ref1] Agardh E, Allebeck P, Hallqvist J, Moradi T and Sidorchuk A (2011) Type 2 diabetes incidence and socio-economic position: A systematic review and meta-analysis. *International Journal of Epidemiology* 40, 804–818. 21335614 10.1093/ije/dyr029

[ref2] Akmatov MK, Ermakova T and Bätzing J (2021) Psychiatric and nonpsychiatric comorbidities among children with ADHD: An exploratory analysis of nationwide claims data in Germany. *Journal of Attention Disorders* 25, 874–884. 31364481 10.1177/1087054719865779

[ref3] Ansari A and Crosnoe R (2016) Children’s hyperactivity, television viewing, and the potential for child effects. *Children and Youth Services Review* 61, 135–140. 26834301 10.1016/j.childyouth.2015.12.018 PMC4730879

[ref4] Baranova A, Chandhoke V, Cao H and Zhang F (2023) Shared genetics and bidirectional causal relationships between type 2 diabetes and attention-deficit/hyperactivity disorder. *General Psychiatry* 36, e100996. 10.1136/gpsych-2022-100996 PMC10016243 36937092

[ref5] Baranova A, Wang J, Cao H, Chen JH, Chen J, Chen M, Ni S, Xu X, Ke X, Xie S, Sun J and Zhang F (2022) Shared genetics between autism spectrum disorder and attention-deficit/hyperactivity disorder and their association with extraversion. *Psychiatry Research* 314, 114679. 10.1016/j.psychres.2022.114679 35717853

[ref6] Bradfield JP, Vogelezang S, Felix JF, Chesi A, Helgeland Ø, Horikoshi M, Karhunen V, Lowry E, Cousminer DL, Ahluwalia TS, Thiering E, Boh ET, Zafarmand MH, Vilor-Tejedor N, Wang CA, Joro R, Chen Z, Gauderman WJ, Pitkänen N, Parra EJ, Fernandez-Rhodes L, Alyass A, Monnereau C, Curtin JA, Have CT, McCormack SE, Hollensted M, Frithioff-Bøjsøe C, Valladares-Salgado A, Peralta-Romero J, Teo YY, Standl M, Leinonen JT, Holm JC, Peters T, Vioque J, Vrijheid M, Simpson A, Custovic A, Vaudel M, Canouil M, Lindi V, Atalay M, Kähönen M, Raitakari OT, van Schaik BDC, Berkowitz RI, Cole SA, Voruganti VS, Wang Y, Highland HM, Comuzzie AG, Butte NF, Justice AE, Gahagan S, Blanco E, Lehtimäki T, Lakka TA, Hebebrand J, Bonnefond A, Grarup N, Froguel P, Lyytikäinen LP, Cruz M, Kobes S, Hanson RL, Zemel BS, Hinney A, Teo KK, Meyre D, North KE, Gilliland FD, Bisgaard H, Bustamante M, Bonnelykke K, Pennell CE, Rivadeneira F, Uitterlinden AG, Baier LJ, Vrijkotte TGM, Heinrich J, Sørensen TIA, Saw SM, Pedersen O, Hansen T, Eriksson J, Widén E, McCarthy MI, Njølstad PR, Power C, Hyppönen E, Sebert S, Brown CD, Järvelin MR, Timpson NJ, Johansson S, Hakonarson H, Jaddoe VWV and Grant SFA (2019) A trans-ancestral meta-analysis of genome-wide association studies reveals loci associated with childhood obesity. *Human Molecular Genetics* 28, 3327–3338. 31504550 10.1093/hmg/ddz161 PMC6859434

[ref7] Breslau J, Miller E, Joanie Chung WJ and Schweitzer JB (2011) Childhood and adolescent onset psychiatric disorders, substance use, and failure to graduate high school on time. *Journal of Psychiatric Research* 45, 295–301. 20638079 10.1016/j.jpsychires.2010.06.014 PMC2962709

[ref8] Brumpton B, Sanderson E, Heilbron K, Hartwig FP, Harrison S, Vie G, Cho Y, Howe LD, Hughes A, Boomsma DI, Havdahl A, Hopper J, Neale M, Nivard MG, Pedersen NL, Reynolds CA, Tucker-Drob EM, Grotzinger A, Howe L, Morris T, Li S, Auton A, Windmeijer F, Chen WM, Bjørngaard JH, Hveem K, Willer C, Evans DM, Kaprio J, Davey Smith G, Åsvold BO, Hemani G and Davies NM (2020) Avoiding dynastic, assortative mating, and population stratification biases in Mendelian randomization through within-family analyses. *Nature Communications* 11, 3519. 10.1038/s41467-020-17117-4 PMC7360778 32665587

[ref9] Burgess S, Butterworth A and Thompson SG (2013) Mendelian randomization analysis with multiple genetic variants using summarized data. *Genetic Epidemiology* 37, 658–665. 24114802 10.1002/gepi.21758 PMC4377079

[ref10] Burgess S, Daniel RM, Butterworth AS and Thompson SG (2015) Network Mendelian randomization: Using genetic variants as instrumental variables to investigate mediation in causal pathways. *International Journal of Epidemiology* 44, 484–495. 25150977 10.1093/ije/dyu176 PMC4469795

[ref11] Burgess S, Davies NM and Thompson SG (2016) Bias due to participant overlap in two-sample Mendelian randomization. *Genetic Epidemiology* 40, 597–608. 27625185 10.1002/gepi.21998 PMC5082560

[ref12] Burgess S and Thompson SG (2015) Multivariable Mendelian randomization: The use of pleiotropic genetic variants to estimate causal effects. *American Journal of Epidemiology* 181, 251–260. 25632051 10.1093/aje/kwu283 PMC4325677

[ref13] Byrne EM, Yang J and Wray NR (2017) Inference in psychiatry via 2-sample Mendelian randomization—From association to causal pathway? *JAMA Psychiatry* 74, 1191–1192. 29094155 10.1001/jamapsychiatry.2017.3162

[ref14] Carstensen B, Rønn PF and Jørgensen ME (2020) Prevalence, incidence and mortality of type 1 and type 2 diabetes in Denmark 1996-2016. *BMJ Open Diabetes Research and Care* 8, e001071. 10.1136/bmjdrc-2019-001071 PMC7265004 32475839

[ref15] Carter AR, Sanderson E, Hammerton G, Richmond RC, Davey Smith G, Heron J, Taylor AE, Davies NM and Howe LD (2021) Mendelian randomisation for mediation analysis: Current methods and challenges for implementation. *European Journal of Epidemiology* 36, 465–478. 33961203 10.1007/s10654-021-00757-1 PMC8159796

[ref16] Cassidy S, Chau JY, Catt M, Bauman A and Trenell MI (2017) Low physical activity, high television viewing and poor sleep duration cluster in overweight and obese adults; a cross-sectional study of 398,984 participants from the UK Biobank. *International Journal of Behavioral Nutrition and Physical Activity* 14, 57. 10.1186/s12966-017-0514-y PMC5408822 28454540

[ref17] Censin JC, Peters SAE, Bovijn J, Ferreira T, Pulit SL, Mägi R, Mahajan A, Holmes MV and Lindgren CM (2019) Causal relationships between obesity and the leading causes of death in women and men. *PLoS Genetics* 15, e1008405. 10.1371/journal.pgen.1008405 PMC6812754 31647808

[ref18] Chen MH, Pan TL, Hsu JW, Huang KL, Su TP, Li CT, Lin WC, Tsai SJ, Chang WH, Chen TJ and Bai YM (2018a) Risk of type 2 diabetes in adolescents and young adults with attention-deficit/hyperactivity disorder: A nationwide longitudinal study. *The Journal of Clinical Psychiatry* 79, 17m11607. 10.4088/JCP.17m11607 29727071

[ref19] Chen Q, Hartman CA, Haavik J, Harro J, Klungsøyr K, Hegvik T-A, Wanders R, Ottosen C, Dalsgaard S, Faraone SV and Larsson H (2018b) Common psychiatric and metabolic comorbidity of adult attention-deficit/hyperactivity disorder: A population-based cross-sectional study. *PLoS One* 13, e0204516. 10.1371/journal.pone.0204516 PMC6157884 30256837

[ref20] Choi JW, Han DH, Kang KD, Jung HY and Renshaw PF (2015) Aerobic exercise and attention deficit hyperactivity disorder: Brain research. *Medicine and Science in Sports and Exercise* 47, 33–39. 24824770 10.1249/MSS.0000000000000373 PMC5504911

[ref21] Cook BG, Li D and Heinrich KM (2015) Obesity, physical activity, and sedentary behavior of youth with learning disabilities and ADHD. *Journal of Learning Disabilities* 48, 563–576. 24449262 10.1177/0022219413518582

[ref22] Cortese S and Castellanos FX (2014) The relationship between ADHD and obesity: Implications for therapy. *Expert Review of Neurotherapeutics* 14, 473–479. 24701972 10.1586/14737175.2014.904748

[ref23] Cortese S, Moreira-Maia CR, St Fleur D, Morcillo-Peñalver C, Rohde LA and Faraone SV (2016) Association between ADHD and obesity: A systematic review and meta-analysis. *American Journal of Psychiatry* 173, 34–43. 26315982 10.1176/appi.ajp.2015.15020266

[ref24] Davies NM, Howe LJ, Brumpton B, Havdahl A, Evans DM and Davey Smith G (2019) Within family Mendelian randomization studies. *Human Molecular Genetics* 28, R170–r179. 31647093 10.1093/hmg/ddz204

[ref25] DeFronzo RA, Ferrannini E, Groop L, Henry RR, Herman WH, Holst JJ, Hu FB, Kahn CR, Raz I, Shulman GI, Simonson DC, Testa MA and Weiss R (2015) Type 2 diabetes mellitus. *Nature Reviews Disease Primers* 1, 15019. 10.1038/nrdp.2015.19 27189025

[ref26] Demontis D, Walters GB, Athanasiadis G, Walters R, Therrien K, Nielsen TT, Farajzadeh L, Voloudakis G, Bendl J, Zeng B, Zhang W, Grove J, Als TD, Duan J, Satterstrom FK, Bybjerg-Grauholm J, Bækved-Hansen M, Gudmundsson OO, Magnusson SH, Baldursson G, Davidsdottir K, Haraldsdottir GS, Agerbo E, Hoffman GE, Dalsgaard S, Martin J, Ribasés M, Boomsma DI, Soler Artigas M, Roth Mota N, Howrigan D, Medland SE, Zayats T, Rajagopal VM, Havdahl A, Doyle A, Reif A, Thapar A, Cormand B, Liao C, Burton C, Bau CHD, Rovaris DL, Sonuga-Barke E, Corfield E, Grevet EH, Larsson H, Gizer IR, Waldman I, Brikell I, Haavik J, Crosbie J, McGough J, Kuntsi J, Glessner J, Langley K, Lesch K-P, Rohde LA, Hutz MH, Klein M, Bellgrove M, Tesli M, O’Donovan MC, Andreassen OA, Leung PWL, Pan PM, Joober R, Schachar R, Loo S, Witt SH, Reichborn-Kjennerud T, Banaschewski T, Hawi Z, Daly MJ, Mors O, Nordentoft M, Mors O, Hougaard DM, Mortensen PB, Daly MJ, Faraone SV, Stefansson H, Roussos P, Franke B, Werge T, Neale BM, Stefansson K, Børglum AD and Consortium AWGotPG and i P-BC (2023) Genome-wide analyses of ADHD identify 27 risk loci, refine the genetic architecture and implicate several cognitive domains. *Nature Genetics* 55, 198–208. 36702997 10.1038/s41588-022-01285-8 PMC10914347

[ref27] Du R, Zhou Y, You C, Liu K, King DA, Liang ZS, Ranson JM, Llewellyn DJ, Huang J and Zhang Z (2023) Attention-deficit/hyperactivity disorder and ischemic stroke: A Mendelian randomization study. *International Journal of Stroke* 18(3), 346–353. 35670701 10.1177/17474930221108272

[ref28] Emdin CA, Anderson SG, Woodward M and Rahimi K (2015) Usual blood pressure and risk of new-onset diabetes. *Journal of the American College of Cardiology* 66, 1552–1562. 26429079 10.1016/j.jacc.2015.07.059 PMC4595710

[ref29] Evangelou E, Warren HR, Mosen-Ansorena D, Mifsud B, Pazoki R, Gao H, Ntritsos G, Dimou N, Cabrera CP, Karaman I, Ng FL, Evangelou M, Witkowska K, Tzanis E, Hellwege JN, Giri A, Velez Edwards DR, Sun YV, Cho K, Gaziano JM, Wilson PWF, Tsao PS, Kovesdy CP, Esko T, Mägi R, Milani L, Almgren P, Boutin T, Debette S, Ding J, Giulianini F, Holliday EG, Jackson AU, Li-Gao R, Lin W-Y, Luan J, Mangino M, Oldmeadow C, Prins BP, Qian Y, Sargurupremraj M, Shah N, Surendran P, Thériault S, Verweij N, Willems SM, Zhao J-H, Amouyel P, Connell J, de Mutsert R, Doney ASF, Farrall M, Menni C, Morris AD, Noordam R, Paré G, Poulter NR, Shields DC, Stanton A, Thom S, Abecasis G, Amin N, Arking DE, Ayers KL, Barbieri CM, Batini C, Bis JC, Blake T, Bochud M, Boehnke M, Boerwinkle E, Boomsma DI, Bottinger EP, Braund PS, Brumat M, Campbell A, Campbell H, Chakravarti A, Chambers JC, Chauhan G, Ciullo M, Cocca M, Collins F, Cordell HJ, Davies G, de Borst MH, de Geus EJ, Deary IJ, Deelen J, Del Greco MF, Demirkale CY, Dörr M, Ehret GB, Elosua R, Enroth S, Erzurumluoglu AM, Ferreira T, Frånberg M, Franco OH, Gandin I, Gasparini P, Giedraitis V, Gieger C, Girotto G, Goel A, Gow AJ, Gudnason V, Guo X, Gyllensten U, Hamsten A, Harris TB, Harris SE, Hartman CA, Havulinna AS, Hicks AA, Hofer E, Hofman A, Hottenga J-J, Huffman JE, Hwang S-J, Ingelsson E, James A, Jansen R, Jarvelin M-R, Joehanes R, Johansson Å, Johnson AD, Joshi PK, Jousilahti P, Jukema JW, Jula A, Kähönen M, Kathiresan S, Keavney BD, Khaw K-T, Knekt P, Knight J, Kolcic I, Kooner JS, Koskinen S, Kristiansson K, Kutalik Z, Laan M, Larson M, Launer LJ, Lehne B, Lehtimäki T, Liewald DCM, Lin L, Lind L, Lindgren CM, Liu Y, Loos RJF, Lopez LM, Lu Y, Lyytikäinen L-P, Mahajan A, Mamasoula C, Marrugat J, Marten J, Milaneschi Y, Morgan A, Morris AP, Morrison AC, Munson PJ, Nalls MA, Nandakumar P, Nelson CP, Niiranen T, Nolte IM, Nutile T, Oldehinkel AJ, Oostra BA, O’Reilly PF, Org E, Padmanabhan S, Palmas W, Palotie A, Pattie A, Penninx BWJH, Perola M, Peters A, Polasek O, Pramstaller PP, Nguyen QT, Raitakari OT, Ren M, Rettig R, Rice K, Ridker PM, Ried JS, Riese H, Ripatti S, Robino A, Rose LM, Rotter JI, Rudan I, Ruggiero D, Saba Y, Sala CF, Salomaa V, Samani NJ, Sarin A-P, Schmidt R, Schmidt H, Shrine N, Siscovick D, Smith AV, Snieder H, Sõber S, Sorice R, Starr JM, Stott DJ, Strachan DP, Strawbridge RJ, Sundström J, Swertz MA, Taylor KD, Teumer A, Tobin MD, Tomaszewski M, Toniolo D, Traglia M, Trompet S, Tuomilehto J, Tzourio C, Uitterlinden AG, Vaez A, van der Most PJ, van Duijn CM, Vergnaud A-C, Verwoert GC, Vitart V, Völker U, Vollenweider P, Vuckovic D, Watkins H, Wild SH, Willemsen G, Wilson JF, Wright AF, Yao J, Zemunik T, Zhang W, Attia JR, Butterworth AS, Chasman DI, Conen D, Cucca F, Danesh J, Hayward C, Howson JMM, Laakso M, Lakatta EG, Langenberg C, Melander O, Mook-Kanamori DO, Palmer CNA, Risch L, Scott RA, Scott RJ, Sever P, Spector TD, van der Harst P, Wareham NJ, Zeggini E, Levy D, Munroe PB, Newton-Cheh C, Brown MJ, Metspalu A, Hung AM, O’Donnell CJ, Edwards TL, Psaty BM, Tzoulaki I, Barnes MR, Wain LV, Elliott P, Caulfield MJ and The Million Veteran P (2018) Genetic analysis of over 1 million people identifies 535 new loci associated with blood pressure traits. *Nature Genetics* 50, 1412–1425. 30224653 10.1038/s41588-018-0205-x PMC6284793

[ref30] Faraone SV, Asherson P, Banaschewski T, Biederman J, Buitelaar JK, Ramos-Quiroga JA, Rohde LA, Sonuga-Barke EJ, Tannock R and Franke B (2015) Attention-deficit/hyperactivity disorder. *Nature Reviews Disease Primers* 1, 15020. 10.1038/nrdp.2015.20 27189265

[ref31] Faraone SV, Biederman J and Mick E (2006) The age-dependent decline of attention deficit hyperactivity disorder: A meta-analysis of follow-up studies. *Psychological Medicine* 36, 159–165. 16420712 10.1017/S003329170500471X

[ref32] Fast K, Björk A, Strandberg M, Johannesson E, Wentz E and Dahlgren J (2021) Half of the children with overweight or obesity and attention-deficit/hyperactivity disorder reach normal weight with stimulants. *Acta Paediatrica (Oslo, Norway: 1992)* 110, 2825–2832. 33876865 10.1111/apa.15881

[ref33] Fleming M, Fitton CA, Steiner MFC, McLay JS, Clark D, King A, Mackay DF and Pell JP (2017) Educational and health outcomes of children treated for attention-deficit/hyperactivity disorder. *JAMA Pediatrics* 171, e170691. 10.1001/jamapediatrics.2017.0691 PMC6583483 28459927

[ref34] Galobardes B, Shaw M, Lawlor DA, Lynch JW and Davey Smith G (2006a) Indicators of socioeconomic position (part 1). *Journal of Epidemiology & Community Health* 60, 7–12. 10.1136/jech.2004.023531 PMC2465546 16361448

[ref35] Galobardes B, Shaw M, Lawlor DA, Lynch JW and Davey Smith G (2006b) Indicators of socioeconomic position (part 2). *Journal of Epidemiology & Community Health* 60, 95–101. 16415256 10.1136/jech.2004.028092 PMC2566160

[ref36] Gao X, Meng LX, Ma KL, Liang J, Wang H, Gao Q and Wang T (2019) The bidirectional causal relationships of insomnia with five major psychiatric disorders: A Mendelian randomization study. *European Psychiatry* 60, 79–85. 31234011 10.1016/j.eurpsy.2019.05.004

[ref37] Garcia-Argibay M, Li L, Du Rietz E, Zhang L, Yao H, Jendle J, Ramos-Quiroga JA, Ribasés M, Chang Z, Brikell I, Cortese S and Larsson H (2023) The association between type 2 diabetes and attention-deficit/hyperactivity disorder: A systematic review, meta-analysis, and population-based sibling study. *Neuroscience and Biobehavioral Reviews* 147, 105076. 10.1016/j.neubiorev.2023.105076 36754221

[ref38] Gavin JR, Rodbard HW, Battelino T, Brosius F, Ceriello A, Cosentino F, Giorgino F, Green J, Ji L, Kellerer M, Koob S, Kosiborod M, Lalic N, Marx N, Prashant Nedungadi T, Parkin CG, Topsever P, Rydén L, Huey-Herng Sheu W, Standl E, Olav Vandvik P and Schnell O (2024) Disparities in prevalence and treatment of diabetes, cardiovascular and chronic kidney diseases – Recommendations from the taskforce of the guideline workshop. *Diabetes Research and Clinical Practice* 211, 111666. 10.1016/j.diabres.2024.111666 38616041

[ref39] Güngör S, Celiloğlu ÖS, Raif SG, Özcan Ö and Selimoğlu MA (2016) Malnutrition and obesity in children with ADHD. *Journal of Attention Disorders* 20, 647–652. 23475827 10.1177/1087054713478465

[ref40] Hartman CA (2020) A solid knowledge base on the seriousness of childhood-onset mental disorders to advance research into causal mechanisms. *JAMA Psychiatry* 77, 783–784. 32211822 10.1001/jamapsychiatry.2019.4908

[ref41] Hemani G, Bowden J, Haycock P, Zheng J, Davis O, Flach P, Gaunt T and Smith GD (2017) Automating Mendelian randomization through machine learning to construct a putative causal map of the human phenome. *bioRxiv* 173682.

[ref42] Hemani G, Zheng J, Elsworth B, Wade KH, Haberland V, Baird D, Laurin C, Burgess S, Bowden J, Langdon R, Tan VY, Yarmolinsky J, Shihab HA, Timpson NJ, Evans DM, Relton C, Martin RM, Davey Smith G, Gaunt TR and Haycock PC (2018) The MR-Base platform supports systematic causal inference across the human phenome. *Elife* 7, e3440. 10.7554/eLife.34408 PMC5976434 29846171

[ref43] Howard AL, Robinson M, Smith GJ, Ambrosini GL, Piek JP and Oddy WH (2011) ADHD is associated with a “Western” dietary pattern in adolescents. *Journal of Attention Disorders* 15, 403–411. 20631199 10.1177/1087054710365990

[ref44] Howe LJ, Lawson DJ, Davies NM, St Pourcain B, Lewis SJ, Davey Smith G and Hemani G (2019) Genetic evidence for assortative mating on alcohol consumption in the UK Biobank. *Nature Communications* 10, 5039. 10.1038/s41467-019-12424-x PMC6864067 31745073

[ref45] Howe LJ, Nivard MG, Morris TT, Hansen AF, Rasheed H, Cho Y, Chittoor G, Ahlskog R, Lind PA, Palviainen T, van der Zee MD, Cheesman R, Mangino M, Wang Y, Li S, Klaric L, Ratliff SM, Bielak LF, Nygaard M, Giannelis A, Willoughby EA, Reynolds CA, Balbona JV, Andreassen OA, Ask H, Baras A, Bauer CR, Boomsma DI, Campbell A, Campbell H, Chen Z, Christofidou P, Corfield E, Dahm CC, Dokuru DR, Evans LM, de Geus EJC, Giddaluru S, Gordon SD, Harden KP, Hill WD, Hughes A, Kerr SM, Kim Y, Kweon H, Latvala A, Lawlor DA, Li L, Lin K, Magnus P, Magnusson PKE, Mallard TT, Martikainen P, Mills MC, Njølstad PR, Overton JD, Pedersen NL, Porteous DJ, Reid J, Silventoinen K, Southey MC, Stoltenberg C, Tucker-Drob EM, Wright MJ, Hewitt JK, Keller MC, Stallings MC, Lee JJ, Christensen K, Kardia SLR, Peyser PA, Smith JA, Wilson JF, Hopper JL, Hägg S, Spector TD, Pingault JB, Plomin R, Havdahl A, Bartels M, Martin NG, Oskarsson S, Justice AE, Millwood IY, Hveem K, Naess Ø, Willer CJ, Åsvold BO, Koellinger PD, Kaprio J, Medland SE, Walters RG, Benjamin DJ, Turley P, Evans DM, Davey Smith G, Hayward C, Brumpton B, Hemani G and Davies NM (2022) Within-sibship genome-wide association analyses decrease bias in estimates of direct genetic effects. *Nature Genetics* 54, 581–592. 35534559 10.1038/s41588-022-01062-7 PMC9110300

[ref46] Huang L, Wang Y, Zhang L, Zheng Z, Zhu T, Qu Y and Mu D (2018) Maternal smoking and attention-deficit/hyperactivity disorder in offspring: A meta-analysis. *Pediatrics* 141, e20172465. 10.1542/peds.2017-2465 29288161

[ref47] Hvolgaard Mikkelsen S, Olsen J, Bech BH and Obel C (2017) Parental age and attention-deficit/hyperactivity disorder (ADHD). *International Journal of Epidemiology* 46, 409–420. 27170763 10.1093/ije/dyw073

[ref48] Jangmo A, Stålhandske A, Chang Z, Chen Q, Almqvist C, Feldman I, Bulik CM, Lichtenstein P, D’Onofrio B, Kuja-Halkola R and Larsson H (2019) Attention-deficit/hyperactivity disorder, school performance, and effect of medication. *Journal of the American Academy of Child & Adolescent Psychiatry* 58, 423–432. 30768391 10.1016/j.jaac.2018.11.014 PMC6541488

[ref49] Jones A, Armstrong B, Weaver RG, Parker H, von Klinggraeff L and Beets MW (2021) Identifying effective intervention strategies to reduce children’s screen time: A systematic review and meta-analysis. *International Journal of Behavioral Nutrition and Physical Activity* 18, 126. 10.1186/s12966-021-01189-6 PMC8447784 34530867

[ref50] Karhunen V, Bond TA, Zuber V, Hurtig T, Moilanen I, Järvelin MR, Evangelou M and Rodriguez A (2021) The link between attention deficit hyperactivity disorder (ADHD) symptoms and obesity-related traits: Genetic and prenatal explanations. *Translational Psychiatry* 11, 455. 10.1038/s41398-021-01584-4 PMC8418601 34482360

[ref51] Keilow M, Holm A and Fallesen P (2018) Medical treatment of attention deficit/hyperactivity disorder (ADHD) and children’s academic performance. *PLoS One* 13, e0207905. 10.1371/journal.pone.0207905 PMC6264851 30496240

[ref52] Kong A, Thorleifsson G, Frigge ML, Vilhjalmsson BJ, Young AI, Thorgeirsson TE, Benonisdottir S, Oddsson A, Halldorsson BV, Masson G, Gudbjartsson DF, Helgason A, Bjornsdottir G, Thorsteinsdottir U and Stefansson K (2018) The nature of nurture: Effects of parental genotypes. *Science* 359, 424–428. 29371463 10.1126/science.aan6877

[ref53] Korrel H, Mueller KL, Silk T, Anderson V and Sciberras E (2017) Research review: Language problems in children with attention-deficit hyperactivity disorder – A systematic meta-analytic review. *Journal of Child Psychology and Psychiatry* 58, 640–654. 28186338 10.1111/jcpp.12688

[ref54] Krishnan S, Cozier YC, Rosenberg L and Palmer JR (2010) Socioeconomic status and incidence of type 2 diabetes: Results from the black women’s health study. *American Journal of Epidemiology* 171, 564–570. 20133518 10.1093/aje/kwp443 PMC2842221

[ref55] Landau Z and Pinhas-Hamiel O (2019) Attention deficit/hyperactivity, the metabolic syndrome, and type 2 diabetes. *Current Diabetes Reports* 19, 46. 10.1007/s11892-019-1174-x 31250219

[ref56] Lee JJ, Wedow R, Okbay A, Kong E, Maghzian O, Zacher M, Nguyen-Viet TA, Bowers P, Sidorenko J, Karlsson Linnér R, Fontana MA, Kundu T, Lee C, Li H, Li R, Royer R, Timshel PN, Walters RK, Willoughby EA, Yengo L, Agee M, Alipanahi B, Auton A, Bell RK, Bryc K, Elson SL, Fontanillas P, Hinds DA, McCreight JC, Huber KE, Litterman NK, McIntyre MH, Mountain JL, Noblin ES, Northover CAM, Pitts SJ, Sathirapongsasuti JF, Sazonova OV, Shelton JF, Shringarpure S, Tian C, Vacic V, Wilson CH, Okbay A, Beauchamp JP, Fontana MA, Lee JJ, Pers TH, Rietveld CA, Turley P, Chen G-B, Emilsson V, Meddens SFW, Oskarsson S, Pickrell JK, Thom K, Timshel P, Vlaming Rd, Abdellaoui A, Ahluwalia TS, Bacelis J, Baumbach C, Bjornsdottir G, Brandsma JH, Concas MP, Derringer J, Furlotte NA, Galesloot TE, Girotto G, Gupta R, Hall LM, Harris SE, Hofer E, Horikoshi M, Huffman JE, Kaasik K, Kalafati IP, Karlsson R, Kong A, Lahti J, van der Lee SJ, Leeuw Cd, Lind PA, Lindgren K-O, Liu T, Mangino M, Marten J, Mihailov E, Miller MB, van der Most PJ, Oldmeadow C, Payton A, Pervjakova N, Peyrot WJ, Qian Y, Raitakari O, Rueedi R, Salvi E, Schmidt B, Schraut KE, Shi J, Smith AV, Poot RA, St Pourcain B, Teumer A, Thorleifsson G, Verweij N, Vuckovic D, Wellmann J, Westra H-J, Yang J, Zhao W, Zhu Z, Alizadeh BZ, Amin N, Bakshi A, Baumeister SE, Biino G, Bønnelykke K, Boyle PA, Campbell H, Cappuccio FP, Davies G, De Neve J-E, Deloukas P, Demuth I, Ding J, Eibich P, Eisele L, Eklund N, Evans DM, Faul JD, Feitosa MF, Forstner AJ, Gandin I, Gunnarsson B, Halldórsson BV, Harris TB, Heath AC, Hocking LJ, Holliday EG, Homuth G, Horan MA, Hottenga J-J, de Jager PL, Joshi PK, Jugessur A, Kaakinen MA, Kähönen M, Kanoni S, Keltigangas-Järvinen L, Kiemeney LALM, Kolcic I, Koskinen S, Kraja AT, Kroh M, Kutalik Z, Latvala A, Launer LJ, Lebreton MP, Levinson DF, Lichtenstein P, Lichtner P, Liewald DCM, Loukola LCSA, Madden PA, Mägi R, Mäki-Opas T, Marioni RE, Marques-Vidal P, Meddens GA, McMahon G, Meisinger C, Meitinger T, Milaneschi Y, Milani L, Montgomery GW, Myhre R, Nelson CP, Nyholt DR, Ollier WER, Palotie A, Paternoster L, Pedersen NL, Petrovic KE, Porteous DJ, Räikkönen K, Ring SM, Robino A, Rostapshova O, Rudan I, Rustichini A, Salomaa V, Sanders AR, Sarin A-P, Schmidt H, Scott RJ, Smith BH, Smith JA, Staessen JA, Steinhagen-Thiessen E, Strauch K, Terracciano A, Tobin MD, Ulivi S, Vaccargiu S, Quaye L, van Rooij FJA, Venturini C, Vinkhuyzen AAE, Völker U, Völzke H, Vonk JM, Vozzi D, Waage J, Ware EB, Willemsen G, Attia JR, Bennett DA, Berger K, Bertram L, Bisgaard H, Boomsma DI, Borecki IB, Bültmann U, Chabris CF, Cucca F, Cusi D, Deary IJ, Dedoussis GV, van Duijn CM, Eriksson JG, Franke B, Franke L, Gasparini P, Gejman PV, Gieger C, Grabe H-J, Gratten J, Groenen PJF, Gudnason V, van der Harst P, Hayward C, Hinds DA, Hoffmann W, Hyppönen E, Iacono WG, Jacobsson B, Järvelin M-R, Jöckel K-H, Kaprio J, Kardia SLR, Lehtimäki T, Lehrer SF, Magnusson PKE, Martin NG, McGue M, Metspalu A, Pendleton N, Penninx BWJH, Perola M, Pirastu N, Pirastu M, Polasek O, Posthuma D, Power C, Province MA, Samani NJ, Schlessinger D, Schmidt R, Sørensen TIA, Spector TD, Stefansson K, Thorsteinsdottir U, Thurik AR, Timpson NJ, Tiemeier H, Tung JY, Uitterlinden AG, Vitart V, Vollenweider P, Weir DR, Wilson JF, Wright AF, Conley DC, Krueger RF, Smith GD, Hofman A, Laibson DI, Medland SE, Meyer MN, Yang J, Johannesson M, Visscher PM, Esko T, Koellinger PD, Cesarini D **and** Me Research T, Cogent and Social Science Genetic Association C (2018) Gene discovery and polygenic prediction from a genome-wide association study of educational attainment in 1.1 million individuals. *Nature Genetics* 50, 1112–1121. 30038396 10.1038/s41588-018-0147-3 PMC6393768

[ref57] Leppert B, Riglin L, Wootton RE, Dardani C, Thapar A, Staley JR, Tilling K, Davey Smith G, Thapar A and Stergiakouli E (2020) The effect of attention deficit/hyperactivity disorder on physical health outcomes: A 2-sample Mendelian randomization study. *American Journal of Epidemiology* 190, 1047–1055. 10.1093/aje/kwaa273 PMC8168225 33324987

[ref58] Li D-D, Yang Y, Gao Z-Y, Zhao L-H, Yang X, Xu F, Yu C, Zhang X-L, Wang X-Q, Wang L-H and Su J-B (2022a) Sedentary lifestyle and body composition in type 2 diabetes. *Diabetology and Metabolic Syndrome* 14, 8. 10.1186/s13098-021-00778-6 PMC8760762 35033170

[ref59] Li GH, Ge GM, Cheung CL, Ip P, Coghill D and Wong IC (2020) Evaluation of causality between ADHD and Parkinson’s disease: Mendelian randomization study. *European Neuropsychopharmacology* 37, 49–63. 32565043 10.1016/j.euroneuro.2020.06.001

[ref60] Li L, Chang Z, Sun J, Garcia-Argibay M, Du Rietz E, Dobrosavljevic M, Brikell I, Jernberg T, Solmi M, Cortese S and Larsson H (2022b) Attention-deficit/hyperactivity disorder as a risk factor for cardiovascular diseases: A nationwide population-based cohort study. *World Psychiatry: Official Journal of the World Psychiatric Association (WPA)* 21, 452–459. 36073682 10.1002/wps.21020 PMC9453905

[ref61] Li L, Yao H, Zhang L, Garcia-Argibay M, Du Rietz E, Brikell I, Solmi M, Cortese S, Ramos-Quiroga JA, Ribasés M, Chang Z and Larsson H (2023) Attention-deficit/hyperactivity disorder is associated with increased risk of cardiovascular diseases: A systematic review and meta-analysis. *JCPP Advances* 3, e12158. 10.1002/jcv2.12158 PMC10501695 37720588

[ref62] Ligthart S, Vaez A, Võsa U, Stathopoulou MG, de Vries PS, Prins BP, Van der Most PJ, Tanaka T, Naderi E, Rose LM, Wu Y, Karlsson R, Barbalic M, Lin H, Pool R, Zhu G, Macé A, Sidore C, Trompet S, Mangino M, Sabater-Lleal M, Kemp JP, Abbasi A, Kacprowski T, Verweij N, Smith AV, Huang T, Marzi C, Feitosa MF, Lohman KK, Kleber ME, Milaneschi Y, Mueller C, Huq M, Vlachopoulou E, Lyytikäinen LP, Oldmeadow C, Deelen J, Perola M, Zhao JH, Feenstra B, Amini M, Lahti J, Schraut KE, Fornage M, Suktitipat B, Chen WM, Li X, Nutile T, Malerba G, Luan J, Bak T, Schork N, Del Greco MF, Thiering E, Mahajan A, Marioni RE, Mihailov E, Eriksson J, Ozel AB, Zhang W, Nethander M, Cheng YC, Aslibekyan S, Ang W, Gandin I, Yengo L, Portas L, Kooperberg C, Hofer E, Rajan KB, Schurmann C, den Hollander W, Ahluwalia TS, Zhao J, Draisma HHM, Ford I, Timpson N, Teumer A, Huang H, Wahl S, Liu Y, Huang J, Uh HW, Geller F, Joshi PK, Yanek LR, Trabetti E, Lehne B, Vozzi D, Verbanck M, Biino G, Saba Y, Meulenbelt I, O’Connell JR, Laakso M, Giulianini F, Magnusson PKE, Ballantyne CM, Hottenga JJ, Montgomery GW, Rivadineira F, Rueedi R, Steri M, Herzig KH, Stott DJ, Menni C, Frånberg M, St Pourcain B, Felix SB, Pers TH, Bakker SJL, Kraft P, Peters A, Vaidya D, Delgado G, Smit JH, Großmann V, Sinisalo J, Seppälä I, Williams SR, Holliday EG, Moed M, Langenberg C, Räikkönen K, Ding J, Campbell H, Sale MM, Chen YI, James AL, Ruggiero D, Soranzo N, Hartman CA, Smith EN, Berenson GS, Fuchsberger C, Hernandez D, Tiesler CMT, Giedraitis V, Liewald D, Fischer K, Mellström D, Larsson A, Wang Y, Scott WR, Lorentzon M, Beilby J, Ryan KA, Pennell CE, Vuckovic D, Balkau B, Concas MP, Schmidt R, Mendes de Leon CF, Bottinger EP, Kloppenburg M, Paternoster L, Boehnke M, Musk AW, Willemsen G, Evans DM, Madden PAF, Kähönen M, Kutalik Z, Zoledziewska M, Karhunen V, Kritchevsky SB, Sattar N, Lachance G, Clarke R, Harris TB, Raitakari OT, Attia JR, van Heemst D, Kajantie E, Sorice R, Gambaro G, Scott RA, Hicks AA, Ferrucci L, Standl M, Lindgren CM, Starr JM, Karlsson M, Lind L, Li JZ, Chambers JC, Mori TA, de Geus E, Heath AC, Martin NG, Auvinen J, Buckley BM, de Craen AJM, Waldenberger M, Strauch K, Meitinger T, Scott RJ, McEvoy M, Beekman M, Bombieri C, Ridker PM, Mohlke KL, Pedersen NL, Morrison AC, Boomsma DI, Whitfield JB, Strachan DP, Hofman A, Vollenweider P, Cucca F, Jarvelin MR, Jukema JW, Spector TD, Hamsten A, Zeller T, Uitterlinden AG, Nauck M, Gudnason V, Qi L, Grallert H, Borecki IB, Rotter JI, März W, Wild PS, Lokki ML, Boyle M, Salomaa V, Melbye M, Eriksson JG, Wilson JF, Penninx B, Becker DM, Worrall BB, Gibson G, Krauss RM, Ciullo M, Zaza G, Wareham NJ, Oldehinkel AJ, Palmer LJ, Murray SS, Pramstaller PP, Bandinelli S, Heinrich J, Ingelsson E, Deary IJ, Mägi R, Vandenput L, van der Harst P, Desch KC, Kooner JS, Ohlsson C, Hayward C, Lehtimäki T, Shuldiner AR, Arnett DK, Beilin LJ, Robino A, Froguel P, Pirastu M, Jess T, Koenig W, Loos RJF, Evans DA, Schmidt H, Smith GD, Slagboom PE, Eiriksdottir G, Morris AP, Psaty BM, Tracy RP, Nolte IM, Boerwinkle E, Visvikis-Siest S, Reiner AP, Gross M, Bis JC, Franke L, Franco OH, Benjamin EJ, Chasman DI, Dupuis J, Snieder H, Dehghan A and Alizadeh BZ (2018) Genome analyses of >200,000 individuals identify 58 loci for chronic inflammation and highlight pathways that link inflammation and complex disorders. *American Journal of Human Genetics* 103, 691–706. 30388399 10.1016/j.ajhg.2018.09.009 PMC6218410

[ref63] Liu CY, Schoeler T, Davies NM, Peyre H, Lim KX, Barker ED, Llewellyn C, Dudbridge F and Pingault JB (2021) Are there causal relationships between attention-deficit/hyperactivity disorder and body mass index? Evidence from multiple genetically informed designs. *International Journal of Epidemiology* 50, 496–509. 33221865 10.1093/ije/dyaa214

[ref64] Liu J, Riesch S, Tien J, Lipman T, Pinto-Martin J and O’Sullivan A (2022) Screen media overuse and associated physical, cognitive, and emotional/behavioral outcomes in children and adolescents: An integrative review. *Journal of Pediatric Health Care* 36, 99–109. 34334279 10.1016/j.pedhc.2021.06.003 PMC10029815

[ref65] Liu M, Jiang Y, Wedow R, Li Y, Brazel DM, Chen F, Datta G, Davila-Velderrain J, McGuire D, Tian C, Zhan X, Agee M, Alipanahi B, Auton A, Bell RK, Bryc K, Elson SL, Fontanillas P, Furlotte NA, Hinds DA, Hromatka BS, Huber KE, Kleinman A, Litterman NK, McIntyre MH, Mountain JL, Northover CAM, Sathirapongsasuti JF, Sazonova OV, Shelton JF, Shringarpure S, Tian C, Tung JY, Vacic V, Wilson CH, Pitts SJ, Mitchell A, Skogholt AH, Winsvold BS, Sivertsen B, Stordal E, Morken G, Kallestad H, Heuch I, Zwart J-A, Fjukstad KK, Pedersen LM, Gabrielsen ME, Johnsen MB, Skrove M, Indredavik MS, Drange OK, Bjerkeset O, Børte S, Stensland SØ, Choquet H, Docherty AR, Faul JD, Foerster JR, Fritsche LG, Gabrielsen ME, Gordon SD, Haessler J, Hottenga J-J, Huang H, Jang S-K, Jansen PR, Ling Y, Mägi R, Matoba N, McMahon G, Mulas A, Orrù V, Palviainen T, Pandit A, Reginsson GW, Skogholt AH, Smith JA, Taylor AE, Turman C, Willemsen G, Young H, Young KA, Zajac GJM, Zhao W, Zhou W, Bjornsdottir G, Boardman JD, Boehnke M, Boomsma DI, Chen C, Cucca F, Davies GE, Eaton CB, Ehringer MA, Esko T, Fiorillo E, Gillespie NA, Gudbjartsson DF, Haller T, Harris KM, Heath AC, Hewitt JK, Hickie IB, Hokanson JE, Hopfer CJ, Hunter DJ, Iacono WG, Johnson EO, Kamatani Y, Kardia SLR, Keller MC, Kellis M, Kooperberg C, Kraft P, Krauter KS, Laakso M, Lind PA, Loukola A, Lutz SM, Madden PAF, Martin NG, McGue M, McQueen MB, Medland SE, Metspalu A, Mohlke KL, Nielsen JB, Okada Y, Peters U, Polderman TJC, Posthuma D, Reiner AP, Rice JP, Rimm E, Rose RJ, Runarsdottir V, Stallings MC, Stančáková A, Stefansson H, Thai KK, Tindle HA, Tyrfingsson T, Wall TL, Weir DR, Weisner C, Whitfield JB, Winsvold BS, Yin J, Zuccolo L, Bierut LJ, Hveem K, Lee JJ, Munafò MR, Saccone NL, Willer CJ, Cornelis MC, David SP, Hinds DA, Jorgenson E, Kaprio J, Stitzel JA, Stefansson K, Thorgeirsson TE, Abecasis G, Liu DJ and Vrieze S and Me Research T and Psychiatry HA-I (2019) Association studies of up to 1.2 million individuals yield new insights into the genetic etiology of tobacco and alcohol use. *Nature Genetics* 51, 237–244. 30643251 10.1038/s41588-018-0307-5 PMC6358542

[ref66] Lu Y, Sjölander A, Cederlöf M, D’Onofrio BM, Almqvist C, Larsson H and Lichtenstein P (2017) Association between medication use and performance on higher education entrance tests in individuals with attention-deficit/hyperactivity disorder. *JAMA Psychiatry* 74, 815–822. 28658471 10.1001/jamapsychiatry.2017.1472 PMC5710548

[ref67] Magliano DJ, Sacre JW, Harding JL, Gregg EW, Zimmet PZ and Shaw JE (2020) Young-onset type 2 diabetes mellitus – Implications for morbidity and mortality. *Nature Reviews Endocrinology* 16, 321–331. 10.1038/s41574-020-0334-z 32203408

[ref68] Mahajan A, Taliun D, Thurner M, Robertson NR, Torres JM, Rayner NW, Payne AJ, Steinthorsdottir V, Scott RA, Grarup N, Cook JP, Schmidt EM, Wuttke M, Sarnowski C, Mägi R, Nano J, Gieger C, Trompet S, Lecoeur C, Preuss MH, Prins BP, Guo X, Bielak LF, Below JE, Bowden DW, Chambers JC, Kim YJ, Ng MCY, Petty LE, Sim X, Zhang W, Bennett AJ, Bork-Jensen J, Brummett CM, Canouil M, Ec Kardt KU, Fischer K, Kardia SLR, Kronenberg F, Läll K, Liu CT, Locke AE, Luan J, Ntalla I, Nylander V, Schönherr S, Schurmann C, Yengo L, Bottinger EP, Brandslund I, Christensen C, Dedoussis G, Florez JC, Ford I, Franco OH, Frayling TM, Giedraitis V, Hackinger S, Hattersley AT, Herder C, Ikram MA, Ingelsson M, Jørgensen ME, Jørgensen T, Kriebel J, Kuusisto J, Ligthart S, Lindgren CM, Linneberg A, Lyssenko V, Mamakou V, Meitinger T, Mohlke KL, Morris AD, Nadkarni G, Pankow JS, Peters A, Sattar N, Stančáková A, Strauch K, Taylor KD, Thorand B, Thorleifsson G, Thorsteinsdottir U, Tuomilehto J, Witte DR, Dupuis J, Peyser PA, Zeggini E, Loos RJF, Froguel P, Ingelsson E, Lind L, Groop L, Laakso M, Collins FS, Jukema JW, Palmer CNA, Grallert H, Metspalu A, Dehghan A, Köttgen A, Abecasis GR, Meigs JB, Rotter JI, Marchini J, Pedersen O, Hansen T, Langenberg C, Wareham NJ, Stefansson K, Gloyn AL, Morris AP, Boehnke M and McCarthy MI (2018) Fine-mapping type 2 diabetes loci to single-variant resolution using high-density imputation and islet-specific epigenome maps. *Nature Genetics* 50, 1505–1513. 30297969 10.1038/s41588-018-0241-6 PMC6287706

[ref69] Martins-Silva T, Vaz JDS, Hutz MH, Salatino-Oliveira A, Genro JP, Hartwig FP, Moreira-Maia CR, Rohde LA, Borges MC and Tovo-Rodrigues L (2019) Assessing causality in the association between attention-deficit/hyperactivity disorder and obesity: A Mendelian randomization study. *International Journal of Obesity* 43, 2500–2508. 31000774 10.1038/s41366-019-0346-8

[ref70] Michaëlsson M, Yuan S, Melhus H, Baron JA, Byberg L, Larsson SC and Michaëlsson K (2022) The impact and causal directions for the associations between diagnosis of ADHD, socioeconomic status, and intelligence by use of a bi-directional two-sample Mendelian randomization design. *BMC Medicine* 20, 106. 10.1186/s12916-022-02314-3 PMC8996513 35399077

[ref71] Millichap JG and Yee MM (2012) The diet factor in attention-deficit/hyperactivity disorder. *Pediatrics* 129, 330–337. 22232312 10.1542/peds.2011-2199

[ref72] Montagni I, Guichard E and Kurth T (2016) Association of screen time with self-perceived attention problems and hyperactivity levels in French students: A cross-sectional study. *BMJ Open* 6, e009089. 10.1136/bmjopen-2015-009089 PMC4769424 26920440

[ref73] Nigg JT, Johnstone JM, Musser ED, Long HG, Willoughby MT and Shannon J (2016) Attention-deficit/hyperactivity disorder (ADHD) and being overweight/obesity: New data and meta-analysis. *Clinical Psychology Review* 43, 67–79. 26780581 10.1016/j.cpr.2015.11.005 PMC4800333

[ref74] Nightingale CM, Rudnicka AR, Donin AS, Sattar N, Cook DG, Whincup PH and Owen CG (2017) Screen time is associated with adiposity and insulin resistance in children. *Archives of Disease in Childhood* 102, 612–616. 28288985 10.1136/archdischild-2016-312016 PMC5519944

[ref75] Nordsletten AE, Larsson H, Crowley JJ, Almqvist C, Lichtenstein P and Mataix-Cols D (2016) Patterns of nonrandom mating within and across 11 major psychiatric disorders. *JAMA Psychiatry* 73, 354–361. 26913486 10.1001/jamapsychiatry.2015.3192 PMC5082975

[ref76] Pelsser LM, Frankena K, Toorman J, Savelkoul HF, Dubois AE, Pereira RR, Haagen TA, Rommelse NN and Buitelaar JK (2011) Effects of a restricted elimination diet on the behaviour of children with attention-deficit hyperactivity disorder (INCA study): A randomised controlled trial. *Lancet* 377, 494–503. 21296237 10.1016/S0140-6736(10)62227-1

[ref77] Pfiffner LJ, Villodas M, Kaiser N, Rooney M and McBurnett K (2013) Educational outcomes of a collaborative school–home behavioral intervention for ADHD. *School Psychology Quarterly* 28, 25. 10.1037/spq0000016 PMC4091627 23506023

[ref78] Polanczyk GV, Willcutt EG, Salum GA, Kieling C and Rohde LA (2014) ADHD prevalence estimates across three decades: An updated systematic review and meta-regression analysis. *International Journal of Epidemiology* 43, 434–442. 24464188 10.1093/ije/dyt261 PMC4817588

[ref79] Quinn PD and D’Onofrio BM (2020) Nature versus nurture. In Benson JB (eds), *Encyclopedia of Infant and Early Childhood Development*, 2nd edn. Oxford: Elsevier, 373–384.

[ref80] Rees JMB, Wood AM and Burgess S (2017) Extending the MR-Egger method for multivariable Mendelian randomization to correct for both measured and unmeasured pleiotropy. *Statistics in Medicine* 36, 4705–4718. 28960498 10.1002/sim.7492 PMC5725762

[ref81] Relton CL and Davey Smith G (2012) Two-step epigenetic Mendelian randomization: A strategy for establishing the causal role of epigenetic processes in pathways to disease. *International Journal of Epidemiology* 41, 161–176. 22422451 10.1093/ije/dyr233 PMC3304531

[ref82] Riglin L and Stergiakouli E (2022) Mendelian randomisation studies of attention deficit hyperactivity disorder. *JCPP Advances* 2, e12117. 10.1002/jcv2.12117 PMC10242846 37431426

[ref83] Roberti JW (2004) A review of behavioral and biological correlates of sensation seeking. *Journal of Research in Personality* 38, 256–279.

[ref84] Rommel AS, Halperin JM, Mill J, Asherson P and Kuntsi J (2013) Protection from genetic diathesis in attention-deficit/hyperactivity disorder: Possible complementary roles of exercise. *Journal of the American Academy of Child & Adolescent Psychiatry* 52, 900–910. 23972692 10.1016/j.jaac.2013.05.018 PMC4257065

[ref85] Ros R and Graziano PA (2018) Social functioning in children with or at risk for attention deficit/hyperactivity disorder: A meta-analytic review. *Journal of Clinical Child & Adolescent Psychology* 47, 213–235. 28128989 10.1080/15374416.2016.1266644

[ref86] Saccaro LF, Schilliger Z, Perroud N and Piguet C (2021) Inflammation, anxiety, and stress in attention-deficit/hyperactivity disorder. *Biomedicines* 9, 1313. 10.3390/biomedicines9101313 PMC8533349 34680430

[ref87] Sanderson E (2021) Multivariable Mendelian randomization and mediation. *Cold Spring Harbor Perspectives in Medicine* 11, a038984. 10.1101/cshperspect.a038984 PMC7849347 32341063

[ref88] Schmengler H, Peeters M, Stevens G, Kunst AE, Hartman CA, Oldehinkel AJ and Vollebergh WAM (2023) Educational level, attention problems, and externalizing behaviour in adolescence and early adulthood: The role of social causation and health-related selection-the TRAILS study. *European Child and Adolescent Psychiatry* 32, 809–824. 34797409 10.1007/s00787-021-01913-4 PMC10147770

[ref89] Selzam S, Ritchie SJ, Pingault JB, Reynolds CA, O’Reilly PF and Plomin R (2019) Comparing within- and between-family polygenic score prediction. *American Journal of Human Genetics* 105, 351–363. 31303263 10.1016/j.ajhg.2019.06.006 PMC6698881

[ref90] Simon V, Czobor P, Bálint S, Mészáros A and Bitter I (2009) Prevalence and correlates of adult attention-deficit hyperactivity disorder: Meta-analysis. *The British Journal of Psychiatry* 194, 204–211. 19252145 10.1192/bjp.bp.107.048827

[ref91] Skrivankova VW, Richmond RC, Woolf BAR, Davies NM, Swanson SA, VanderWeele TJ, Timpson NJ, Higgins JPT, Dimou N, Langenberg C, Loder EW, Golub RM, Egger M, Davey Smith G and Richards JB (2021a) Strengthening the reporting of observational studies in epidemiology using Mendelian randomisation (STROBE-MR): Explanation and elaboration. *BMJ* 375, n2233. 10.1136/bmj.n2233 PMC8546498 34702754

[ref92] Skrivankova VW, Richmond RC, Woolf BAR, Yarmolinsky J, Davies NM, Swanson SA, VanderWeele TJ, Higgins JPT, Timpson NJ, Dimou N, Langenberg C, Golub RM, Loder EW, Gallo V, Tybjaerg-Hansen A, Davey Smith G, Egger M and Richards JB (2021b) Strengthening the reporting of observational studies in epidemiology using Mendelian randomization: The STROBE-MR statement. *JAMA* 326, 1614–1621. 34698778 10.1001/jama.2021.18236

[ref93] Smith GD and Ebrahim S (2003) ‘Mendelian randomization’: Can genetic epidemiology contribute to understanding environmental determinants of disease? *International Journal of Epidemiology* 32, 1–22. 12689998 10.1093/ije/dyg070

[ref94] Smith GD and Ebrahim S (2004) Mendelian randomization: Prospects, potentials, and limitations. *International Journal of Epidemiology* 33, 30–42. 15075143 10.1093/ije/dyh132

[ref95] Soler Artigas M, Sánchez-Mora C, Rovira P, Vilar-Ribó L, Ramos-Quiroga JA and Ribasés M (2023) Mendelian randomization analysis for attention deficit/hyperactivity disorder: Studying a broad range of exposures and outcomes. *International Journal of Epidemiology* 52, 386–402. 35690959 10.1093/ije/dyac128 PMC10114062

[ref96] Sun H, Saeedi P, Karuranga S, Pinkepank M, Ogurtsova K, Duncan BB, Stein C, Basit A, Chan JCN, Mbanya JC, Pavkov ME, Ramachandaran A, Wild SH, James S, Herman WH, Zhang P, Bommer C, Kuo S, Boyko EJ and Magliano DJ (2022) IDF Diabetes Atlas: Global, regional and country-level diabetes prevalence estimates for 2021 and projections for 2045. *Diabetes Research and Clinical Practice* 183, 109119. 10.1016/j.diabres.2021.109119 PMC11057359 34879977

[ref97] Team RC (2014) R: A language and environment for statistical computing. *MSOR Connections* 1.

[ref98] Thomas R, Sanders S, Doust J, Beller E and Glasziou P (2015) Prevalence of attention-deficit/hyperactivity disorder: A systematic review and meta-analysis. *Pediatrics* 135, e994–1001. 25733754 10.1542/peds.2014-3482

[ref99] Treur JL, Demontis D, Smith GD, Sallis H, Richardson TG, Wiers RW, Børglum AD, Verweij KJH and Munafò MR (2021) Investigating causality between liability to ADHD and substance use, and liability to substance use and ADHD risk, using Mendelian randomization. *Addiction Biology* 26, e12849. 10.1111/adb.12849 PMC7228854 31733098

[ref100] UNESCO Institute for Statistics (2012) International standard classification of education. http://uis.unesco.org/sites/default/files/documents/international-standard-classification-of-education-isced-2011-en.pdf (accessed 19 June 2023).

[ref101] Vaidyanathan S, Manohar H, Chandrasekaran V and Kandasamy P (2021) Screen time exposure in preschool children with ADHD: A cross-sectional exploratory study from South India. *Indian Journal of Psychological Medicine* 43, 125–129. 34376887 10.1177/0253717620939782 PMC8313458

[ref102] Vanderweele TJ and Vansteelandt S (2010) Odds ratios for mediation analysis for a dichotomous outcome. *American Journal of Epidemiology* 172, 1339–1348. 21036955 10.1093/aje/kwq332 PMC2998205

[ref103] van de Vegte YJ, Said MA, Rienstra M, van der Harst P and Verweij N (2020) Genome-wide association studies and Mendelian randomization analyses for leisure sedentary behaviours. *Nature Communications* 11, 1770. 10.1038/s41467-020-15553-w PMC7174427 32317632

[ref104] Vandewater EA, Lee JH and Shim M-S (2005) Family conflict and violent electronic media use in school-aged children. *Media Psychology* 7, 73–86.

[ref105] Viner R, White B and Christie D (2017) Type 2 diabetes in adolescents: A severe phenotype posing major clinical challenges and public health burden. *Lancet* 389, 2252–2260. 28589895 10.1016/S0140-6736(17)31371-5

[ref106] Vogelezang S, Bradfield JP, Ahluwalia TS, Curtin JA, Lakka TA, Grarup N, Scholz M, van der Most PJ, Monnereau C, Stergiakouli E, Heiskala A, Horikoshi M, Fedko IO, Vilor-Tejedor N, Cousminer DL, Standl M, Wang CA, Viikari J, Geller F, Íñiguez C, Pitkänen N, Chesi A, Bacelis J, Yengo L, Torrent M, Ntalla I, Helgeland Ø, Selzam S, Vonk JM, Zafarmand MH, Heude B, Farooqi IS, Alyass A, Beaumont RN, Have CT, Rzehak P, Bilbao JR, Schnurr TM, Barroso I, Bønnelykke K, Beilin LJ, Carstensen L, Charles MA, Chawes B, Clément K, Closa-Monasterolo R, Custovic A, Eriksson JG, Escribano J, Groen-Blokhuis M, Grote V, Gruszfeld D, Hakonarson H, Hansen T, Hattersley AT, Hollensted M, Hottenga JJ, Hyppönen E, Johansson S, Joro R, Kähönen M, Karhunen V, Kiess W, Knight BA, Koletzko B, Kühnapfel A, Landgraf K, Langhendries JP, Lehtimäki T, Leinonen JT, Li A, Lindi V, Lowry E, Bustamante M, Medina-Gomez C, Melbye M, Michaelsen KF, Morgen CS, Mori TA, Nielsen TRH, Niinikoski H, Oldehinkel AJ, Pahkala K, Panoutsopoulou K, Pedersen O, Pennell CE, Power C, Reijneveld SA, Rivadeneira F, Simpson A, Sly PD, Stokholm J, Teo KK, Thiering E, Timpson NJ, Uitterlinden AG, van Beijsterveldt CEM, van Schaik BDC, Vaudel M, Verduci E, Vinding RK, Vogel M, Zeggini E, Sebert S, Lind MV, Brown CD, Santa-Marina L, Reischl E, Frithioff-Bøjsøe C, Meyre D, Wheeler E, Ong K, Nohr EA, Vrijkotte TGM, Koppelman GH, Plomin R, Njølstad PR, Dedoussis GD, Froguel P, Sørensen TIA, Jacobsson B, Freathy RM, Zemel BS, Raitakari O, Vrijheid M, Feenstra B, Lyytikäinen LP, Snieder H, Kirsten H, Holt PG, Heinrich J, Widén E, Sunyer J, Boomsma DI, Järvelin MR, Körner A, Davey Smith G, Holm JC, Atalay M, Murray C, Bisgaard H, McCarthy MI, Jaddoe VWV, Grant SFA and Felix JF (2020) Novel loci for childhood body mass index and shared heritability with adult cardiometabolic traits. *PLoS Genetics* 16, e1008718. 10.1371/journal.pgen.1008718 PMC7581004 33045005

[ref107] Vos T, Lim SS, Abbafati C, Abbas KM, Abbasi M, Abbasifard M, Abbasi-Kangevari M, Abbastabar H, Abd-Allah F, Abdelalim A, Abdollahi M, Abdollahpour I, Abolhassani H, Aboyans V, Abrams EM, Abreu LG, Abrigo MRM, Abu-Raddad LJ, Abushouk AI, Acebedo A, Ackerman IN, Adabi M, Adamu AA, Adebayo OM, Adekanmbi V, Adelson JD, Adetokunboh OO, Adham D, Afshari M, Afshin A, Agardh EE, Agarwal G, Agesa KM, Aghaali M, Aghamir SMK, Agrawal A, Ahmad T, Ahmadi A, Ahmadi M, Ahmadieh H, Ahmadpour E, Akalu TY, Akinyemi RO, Akinyemiju T, Akombi B, Al-Aly Z, Alam K, Alam N, Alam S, Alam T, Alanzi TM, Albertson SB, Alcalde-Rabanal JE, Alema NM, Ali M, Ali S, Alicandro G, Alijanzadeh M, Alinia C, Alipour V, Aljunid SM, Alla F, Allebeck P, Almasi-Hashiani A, Alonso J, Al-Raddadi RM, Altirkawi KA, Alvis-Guzman N, Alvis-Zakzuk NJ, Amini S, Amini-Rarani M, Aminorroaya A, Amiri F, Amit AML, Amugsi DA, Amul GGH, Anderlini D, Andrei CL, Andrei T, Anjomshoa M, Ansari F, Ansari I, Ansari-Moghaddam A, Antonio CAT, Antony CM, Antriyandarti E, Anvari D, Anwer R, Arabloo J, Arab-Zozani M, Aravkin AY, Ariani F, Ärnlöv J, Aryal KK, Arzani A, Asadi-Aliabadi M, Asadi-Pooya AA, Asghari B, Ashbaugh C, Atnafu DD, Atre SR, Ausloos F, Ausloos M, Ayala Quintanilla BP, Ayano G, Ayanore MA, Aynalem YA, Azari S, Azarian G, Azene ZN, Babaee E, Badawi A, Bagherzadeh M, Bakhshaei MH, Bakhtiari A, Balakrishnan S, Balalla S, Balassyano S, Banach M, Banik PC, Bannick MS, Bante AB, Baraki AG, Barboza MA, Barker-Collo SL, Barthelemy CM, Barua L, Barzegar A, Basu S, Baune BT, Bayati M, Bazmandegan G, Bedi N, Beghi E, Béjot Y, Bello AK, Bender RG, Bennett DA, Bennitt FB, Bensenor IM, Benziger CP, Berhe K, Bernabe E, Bertolacci GJ, Bhageerathy R, Bhala N, Bhandari D, Bhardwaj P, Bhattacharyya K, Bhutta ZA, Bibi S, Biehl MH, Bikbov B, Bin Sayeed MS, Biondi A, Birihane BM, Bisanzio D, Bisignano C, Biswas RK, Bohlouli S, Bohluli M, Bolla SRR, Boloor A, Boon-Dooley AS, Borges G, Borzì AM, Bourne R, Brady OJ, Brauer M, Brayne C, Breitborde NJK, Brenner H, Briant PS, Briggs AM, Briko NI, Britton GB, Bryazka D, Buchbinder R, Bumgarner BR, Busse R, Butt ZA, Caetano dos Santos FL, Cámera LLAA, Campos-Nonato IR, Car J, Cárdenas R, Carreras G, Carrero JJ, Carvalho F, Castaldelli-Maia JM, Castañeda-Orjuela CA, Castelpietra G, Castle CD, Castro F, Catalá-López F, Causey K, Cederroth CR, Cercy KM, Cerin E, Chandan JS, Chang AR, Charlson FJ, Chattu VK, Chaturvedi S, Chimed-Ochir O, Chin KL, Cho DY, Christensen H, Chu D-T, Chung MT, Cicuttini FM, Ciobanu LG, Cirillo M, Collins EL, Compton K, Conti S, Cortesi PA, Costa VM, Cousin E, Cowden RG, Cowie BC, Cromwell EA, Cross DH, Crowe CS, Cruz JA, Cunningham M, Dahlawi SMA, Damiani G, Dandona L, Dandona R, Darwesh AM, Daryani A, Das JK, Das Gupta R, das Neves J, Dávila-Cervantes CA, Davletov K, De Leo D, Dean FE, DeCleene NK, Deen A, Degenhardt L, Dellavalle RP, Demeke FM, Demsie DG, Denova-Gutiérrez E, Dereje ND, Dervenis N, Desai R, Desalew A, Dessie GA, Dharmaratne SD, Dhungana GP, Dianatinasab M, Diaz D, Dibaji Forooshani ZS, Dingels ZV, Dirac MA, Djalalinia S, Do HT, Dokova K, Dorostkar F, Doshi CP, Doshmangir L, Douiri A, Doxey MC, Driscoll TR, Dunachie SJ, Duncan BB, Duraes AR, Eagan AW, Ebrahimi Kalan M, Edvardsson D, Ehrlich JR, El Nahas N, El Sayed I, El Tantawi M, Elbarazi I, Elgendy IY, Elhabashy HR, El-Jaafary SI, Elyazar IRF, Emamian MH, Emmons-Bell S, Erskine HE, Eshrati B, Eskandarieh S, Esmaeilnejad S, Esmaeilzadeh F, Esteghamati A, Estep K, Etemadi A, Etisso AE, Farahmand M, Faraj A, Fareed M, Faridnia R, Farinha CSeS, Farioli A, Faro A, Faruque M, Farzadfar F, Fattahi N, Fazlzadeh M, Feigin VL, Feldman R, Fereshtehnejad S-M, Fernandes E, Ferrari AJ, Ferreira ML, Filip I, Fischer F, Fisher JL, Fitzgerald R, Flohr C, Flor LS, Foigt NA, Folayan MO, Force LM, Fornari C, Foroutan M, Fox JT, Freitas M, Fu W, Fukumoto T, Furtado JM, Gad MM, Gakidou E, Galles NC, Gallus S, Gamkrelidze A, Garcia-Basteiro AL, Gardner WM, Geberemariyam BS, Gebrehiwot AM, Gebremedhin KB, Gebreslassie AAAA, Gershberg Hayoon A, Gething PW, Ghadimi M, Ghadiri K, Ghafourifard M, Ghajar A, Ghamari F, Ghashghaee A, Ghiasvand H, Ghith N, Gholamian A, Gilani SA, Gill PS, Gitimoghaddam M, Giussani G, Goli S, Gomez RS, Gopalani SV, Gorini G, Gorman TM, Gottlich HC, Goudarzi H, Goulart AC, Goulart BNG, Grada A, Grivna M, Grosso G, Gubari MIM, Gugnani HC, Guimaraes ALS, Guimarães RA, Guled RA, Guo G, Guo Y, Gupta R, Haagsma JA, Haddock B, Hafezi-Nejad N, Hafiz A, Hagins H, Haile LM, Hall BJ, Halvaei I, Hamadeh RR, Hamagharib Abdullah K, Hamilton EB, Han C, Han H, Hankey GJ, Haro JM, Harvey JD, Hasaballah AI, Hasanzadeh A, Hashemian M, Hassanipour S, Hassankhani H, Havmoeller RJ, Hay RJ, Hay SI, Hayat K, Heidari B, Heidari G, Heidari-Soureshjani R, Hendrie D, Henrikson HJ, Henry NJ, Herteliu C, Heydarpour F, Hird TR, Hoek HW, Hole MK, Holla R, Hoogar P, Hosgood HD, Hosseinzadeh M, Hostiuc M, Hostiuc S, Househ M, Hoy DG, Hsairi M, Hsieh VC-r, Hu G, Huda TM, Hugo FN, Huynh CK, Hwang B-F, Iannucci VC, Ibitoye SE, Ikuta KS, Ilesanmi OS, Ilic IM, Ilic MD, Inbaraj LR, Ippolito H, Irvani SSN, Islam MM, Islam M, Islam SMS, Islami F, Iso H, Ivers RQ, Iwu CCD, Iyamu IO, Jaafari J, Jacobsen KH, Jadidi-Niaragh F, Jafari H, Jafarinia M, Jahagirdar D, Jahani MA, Jahanmehr N, Jakovljevic M, Jalali A, Jalilian F, James SL, Janjani H, Janodia MD, Jayatilleke AU, Jeemon P, Jenabi E, Jha RP, Jha V, Ji JS, Jia P, John O, John-Akinola YO, Johnson CO, Johnson SC, Jonas JB, Joo T, Joshi A, Jozwiak JJ, Jürisson M, Kabir A, Kabir Z, Kalani H, Kalani R, Kalankesh LR, Kalhor R, Kamiab Z, Kanchan T, Karami Matin B, Karch A, Karim MA, Karimi SE, Kassa GM, Kassebaum NJ, Katikireddi SV, Kawakami N, Kayode GA, Keddie SH, Keller C, Kereselidze M, Khafaie MA, Khalid N, Khan M, Khatab K, Khater MM, Khatib MN, Khayamzadeh M, Khodayari MT, Khundkar R, Kianipour N, Kieling C, Kim D, Kim Y-E, Kim YJ, Kimokoti RW, Kisa A, Kisa S, Kissimova-Skarbek K, Kivimäki M, Kneib CJ, Knudsen AKS, Kocarnik JM, Kolola T, Kopec JA, Kosen S, Koul PA, Koyanagi A, Kravchenko MA, Krishan K, Krohn KJ, Kuate Defo B, Kucuk Bicer B, Kumar GA, Kumar M, Kumar P, Kumar V, Kumaresh G, Kurmi OP, Kusuma D, Kyu HH, La Vecchia C, Lacey B, Lal DK, Lalloo R, Lam JO, Lami FH, Landires I, Lang JJ, Lansingh VC, Larson SL, Larsson AO, Lasrado S, Lassi ZS, Lau KM-M, Lavados PM, Lazarus JV, Ledesma JR, Lee PH, Lee SWH, LeGrand KE, Leigh J, Leonardi M, Lescinsky H, Leung J, Levi M, Lewington S, Li S, Lim L-L, Lin C, Lin R-T, Linehan C, Linn S, Liu H-C, Liu S, Liu Z, Looker KJ, Lopez AD, Lopukhov PD, Lorkowski S, Lotufo PA, Lucas TCD, Lugo A, Lunevicius R, Lyons RA, Ma J, MacLachlan JH, Maddison ER, Maddison R, Madotto F, Mahasha PW, Mai HT, Majeed A, Maled V, Maleki S, Malekzadeh R, Malta DC, Mamun AA, Manafi A, Manafi N, Manguerra H, Mansouri B, Mansournia MA, Mantilla Herrera AM, Maravilla JC, Marks A, Martins-Melo FR, Martopullo I, Masoumi SZ, Massano J, Massenburg BB, Mathur MR, Maulik PK, McAlinden C, McGrath JJ, McKee M, Mehndiratta MM, Mehri F, Mehta KM, Meitei WB, Memiah PTN, Mendoza W, Menezes RG, Mengesha EW, Mengesha MB, Mereke A, Meretoja A, Meretoja TJ, Mestrovic T, Miazgowski B, Miazgowski T, Michalek IM, Mihretie KM, Miller TR, Mills EJ, Mirica A, Mirrakhimov EM, Mirzaei H, Mirzaei M, Mirzaei-Alavijeh M, Misganaw AT, Mithra P, Moazen B, Moghadaszadeh M, Mohamadi E, Mohammad DK, Mohammad Y, Mohammad Gholi Mezerji N, Mohammadian-Hafshejani A, Mohammadifard N, Mohammadpourhodki R, Mohammed S, Mokdad AH, Molokhia M, Momen NC, Monasta L, Mondello S, Mooney MD, Moosazadeh M, Moradi G, Moradi M, Moradi-Lakeh M, Moradzadeh R, Moraga P, Morales L, Morawska L, Moreno Velásquez I, Morgado-da-Costa J, Morrison SD, Mosser JF, Mouodi S, Mousavi SM, Mousavi Khaneghah A, Mueller UO, Munro SB, Muriithi MK, Musa KI, Muthupandian S, Naderi M, Nagarajan AJ, Nagel G, Naghshtabrizi B, Nair S, Nandi AK, Nangia V, Nansseu JR, Nayak VC, Nazari J, Negoi I, Negoi RI, Netsere HBN, Ngunjiri JW, Nguyen CT, Nguyen J, Nguyen M, Nichols E, Nigatu D, Nigatu YT, Nikbakhsh R, Nixon MR, Nnaji CA, Nomura S, Norrving B, Noubiap JJ, Nowak C, Nunez-Samudio V, Oţoiu A, Oancea B, Odell CM, Ogbo FA, Oh I-H, Okunga EW, Oladnabi M, Olagunju AT, Olusanya BO, Olusanya JO, Oluwasanu MM, Omar Bali A, Omer MO, Ong KL, Onwujekwe OE, Orji AU, Orpana HM, Ortiz A, Ostroff SM, Otstavnov N, Otstavnov SS, Øverland S, Owolabi MO, P A M, Padubidri JR, Pakhare AP, Palladino R, Pana A, Panda-Jonas S, Pandey A, Park E-K, Parmar PGK, Pasupula DK, Patel SK, Paternina-Caicedo AJ, Pathak A, Pathak M, Patten SB, Patton GC, Paudel D, Pazoki Toroudi H, Peden AE, Pennini A, Pepito VCF, Peprah EK, Pereira A, Pereira DM, Perico N, Pham HQ, Phillips MR, Pigott DM, Pilgrim T, Pilz TM, Pirsaheb M, Plana-Ripoll O, Plass D, Pokhrel KN, Polibin RV, Polinder S, Polkinghorne KR, Postma MJ, Pourjafar H, Pourmalek F, Pourmirza Kalhori R, Pourshams A, Poznańska A, Prada SI, Prakash V, Pribadi DRA, Pupillo E, Quazi Syed Z, Rabiee M, Rabiee N, Radfar A, Rafiee A, Rafiei A, Raggi A, Rahimi-Movaghar A, Rahman MA, Rajabpour-Sanati A, Rajati F, Ramezanzadeh K, Ranabhat CL, Rao PC, Rao SJ, Rasella D, Rastogi P, Rathi P, Rawaf DL, Rawaf S, Rawal L, Razo C, Redford SB, Reiner RC, Reinig N, Reitsma MB, Remuzzi G, Renjith V, Renzaho AMN, Resnikoff S, Rezaei N, Rezai Ms, Rezapour A, Rhinehart P-A, Riahi SM, Ribeiro ALP, Ribeiro DC, Ribeiro D, Rickard J, Roberts NLS, Roberts S, Robinson SR, Roever L, Rolfe S, Ronfani L, Roshandel G, Roth GA, Rubagotti E, Rumisha SF, Sabour S, Sachdev PS, Saddik B, Sadeghi E, Sadeghi M, Saeidi S, Safi S, Safiri S, Sagar R, Sahebkar A, Sahraian MA, Sajadi SM, Salahshoor MR, Salamati P, Salehi Zahabi S, Salem H, Salem MRR, Salimzadeh H, Salomon JA, Salz I, Samad Z, Samy AM, Sanabria J, Santomauro DF, Santos IS, Santos JV, Santric-Milicevic MM, Saraswathy SYI, Sarmiento-Suárez R, Sarrafzadegan N, Sartorius B, Sarveazad A, Sathian B, Sathish T, Sattin D, Sbarra AN, Schaeffer LE, Schiavolin S, Schmidt MI, Schutte AE, Schwebel DC, Schwendicke F, Senbeta AM, Senthilkumaran S, Sepanlou SG, Shackelford KA, Shadid J, Shahabi S, Shaheen AA, Shaikh MA, Shalash AS, Shams-Beyranvand M, Shamsizadeh M, Shannawaz M, Sharafi K, Sharara F, Sheena BS, Sheikhtaheri A, Shetty RS, Shibuya K, Shiferaw WS, Shigematsu M, Shin JI, Shiri R, Shirkoohi R, Shrime MG, Shuval K, Siabani S, Sigfusdottir ID, Sigurvinsdottir R, Silva JP, Simpson KE, Singh A, Singh JA, Skiadaresi E, Skou ST, Skryabin VY, Sobngwi E, Sokhan A, Soltani S, Sorensen RJD, Soriano JB, Sorrie MB, Soyiri IN, Sreeramareddy CT, Stanaway JD, Stark BA, Ştefan SC, Stein C, Steiner C, Steiner TJ, Stokes MA, Stovner LJ, Stubbs JL, Sudaryanto A, Sufiyan MaB, Sulo G, Sultan I, Sykes BL, Sylte DO, Szócska M, Tabarés-Seisdedos R, Tabb KM, Tadakamadla SK, Taherkhani A, Tajdini M, Takahashi K, Taveira N, Teagle WL, Teame H, Tehrani-Banihashemi A, Teklehaimanot BF, Terrason S, Tessema ZT, Thankappan KR, Thomson AM, Tohidinik HR, Tonelli M, Topor-Madry R, Torre AE, Touvier M, Tovani-Palone MRR, Tran BX, Travillian R, Troeger CE, Truelsen TC, Tsai AC, Tsatsakis A, Tudor Car L, Tyrovolas S, Uddin R, Ullah S, Undurraga EA, Unnikrishnan B, Vacante M, Vakilian A, Valdez PR, Varughese S, Vasankari TJ, Vasseghian Y, Venketasubramanian N, Violante FS, Vlassov V, Vollset SE, Vongpradith A, Vukovic A, Vukovic R, Waheed Y, Walters MK, Wang J, Wang Y, Wang Y-P, Ward JL, Watson A, Wei J, Weintraub RG, Weiss DJ, Weiss J, Westerman R, Whisnant JL, Whiteford HA, Wiangkham T, Wiens KE, Wijeratne T, Wilner LB, Wilson S, Wojtyniak B, Wolfe CDA, Wool EE, Wulf A-M, Hanson S, Wunrow HY, Xu G, Xu R, Yadgir S, Yahyazadeh Jabbari SH, Yamagishi K, Yaminfirooz M, Yano Y, Yaya S, Yazdi-Feyzabadi V, Yearwood JA, Yeheyis TY, Yeshitila YG, Yip P, Yonemoto N, Yoon S-J, Yoosefi Lebni J, Younis MZ, Younker TP, Yousefi Z, Yousefifard M, Yousefinezhadi T, Yousuf AY, Yu C, Yusefzadeh H, Zahirian Moghadam T, Zaki L, Zaman SB, Zamani M, Zamanian M, Zandian H, Zangeneh A, Zastrozhin MS, Zewdie KA, Zhang Y, Zhang Z-J, Zhao JT, Zhao Y, Zheng P, Zhou M, Ziapour A, Zimsen SRM, Naghavi M and Murray CJL (2020) Global burden of 369 diseases and injuries in 204 countries and territories, 1990-2019: A systematic analysis for the Global Burden of Disease Study 2019. *Lancet* 396, 1204–1222. 33069326 10.1016/S0140-6736(20)30925-9 PMC7567026

[ref108] Wang X, Bao W, Liu J, OuYang Y-Y, Wang D, Rong S, Xiao X, Shan Z-L, Zhang Y, Yao P and Liu L-G (2012) Inflammatory markers and risk of type 2 diabetes: A systematic review and meta-analysis. *Diabete Care* 36, 166–175. 10.2337/dc12-0702 PMC3526249 23264288

[ref109] Yang A, Rolls ET, Dong G, Du J, Li Y, Feng J, Cheng W and Zhao XM (2022) Longer screen time utilization is associated with the polygenic risk for attention-deficit/hyperactivity disorder with mediation by brain white matter microstructure. *EBioMedicine* 80, 104039. 10.1016/j.ebiom.2022.104039 PMC9079003 35509143

[ref110] Yengo L, Sidorenko J, Kemper KE, Zheng Z, Wood AR, Weedon MN, Frayling TM, Hirschhorn J, Yang J and Visscher PM and Consortium tG (2018) Meta-analysis of genome-wide association studies for height and body mass index in ∼700000 individuals of European ancestry. *Human Molecular Genetics* 27, 3641–3649. 30124842 10.1093/hmg/ddy271 PMC6488973

[ref111] Zhang J, Chen Z, Pärna K, van Zon SKR, Snieder H and Thio CHL (2022) Mediators of the association between educational attainment and type 2 diabetes mellitus: A two-step multivariable Mendelian randomisation study. *Diabetologia* 65, 1364–1374. 35482055 10.1007/s00125-022-05705-6 PMC9283137

