# Supplementary material for: Mediating pathways between attention deficit hyperactivity disorder and type 2 diabetes mellitus: evidence from a two-step and multivariable Mendelian randomization study
Source: Epidemiol Psychiatr Sci. 2024 Oct 28;33:e54. doi: 10.1017/S2045796024000593 (PMC11561680; doi:10.1017/S2045796024000593)
Supplement: Zhang et al. supplementary material 2 — Zhang et al. supplementary material [file S2045796024000593sup002.docx]

Mediating pathways between attention deficit hyperactivity disorder and type 2 diabetes mellitus: evidence from a two-step and multivariable Mendelian randomization study

Electronic supplementary material (ESM)

**ESM Methods.** Mendelian randomization (MR) assumptions and methods

**ESM Data.** SNP data **(.xlsx)**

**ESM Figure 1**. MVMR estimates of proportion mediated by each individual mediator using the difference method

**ESM Figure 2**. Reverse Mendelian randomization scatterplots (mediators to ADHD)

**ESM References**

**ESM Methods.** Mendelian randomization assumptions and methods

Causal inference in traditional observational epidemiological studies is limited due to confounding and reverse causation. MR is a method that can be used to uncover causal relationships between exposure and outcome in the presence of such limitations (Lawlor *et al.*, 2008). MR uses SNPs to genetically predict exposures. MR estimates are unconfounded, and thus valid estimates of causality, under a number of key assumptions. The first is the relevance assumption, which assumes that the genetic instruments are strongly associated with the exposure; this assumption is satisfied by the selection of SNPs with robust genome-wide significant (P<5x10^-8^) and replicated associations, while in addition SNP data from both exposure and outcome GWAS are derived from samples of the same underlying target population, in the present case, general European ancestry. The second is the independence assumption, which requires that genetic instruments are not associated with any confounder of the relationship between exposure and outcome; this is assumed to be true due to Mendel’s law of independent segregation, in which genetic variants for a certain trait are inherited independently of other traits. The third is the exclusion restriction criterion, which requires that any effect of the genetic instrument on the outcome variable is solely through the exposure variable. This assumption might be violated due to horizontal pleiotropy, in which a genetic instrument might have direct effects on both the exposure and the outcome. The multivariable Mendelian randomization (MVMR) analysis requires similar assumptions: Firstly, the exposures must be strongly predicted by the SNPs given the other exposures included in the model; Secondly, the SNPs must be independent of the outcome Y given all of the exposures included in the model; Thirdly, the SNPs independent of all confounders of any of the exposures and the outcome Y.

To assess robustness of our results against horizontal pleiotropy, we performed sensitivity analyses. Firstly, we assessed the heterogeneity of Wald ratios (i.e. single SNP MR effect estimates) to find evidence of potential pleiotropy; large heterogeneity in Wald ratios is suggestive of horizontal pleiotropy (**ESM Table 1**). Second, we examined MR funnel plots of Wald ratios; asymmetry in the funnel plots is suggestive of directional horizontal pleiotropy (data not shown). Third, we examined the Egger intercept; significant deviation from a zero intercept is suggestive of directional horizontal pleiotropy. Finally, we conducted sensitivity MR analyses that relax the exclusion restriction criterion using the MOE framework, such as: a) Mendelian randomization-Egger (MR-Egger) (Schmidt & Dudbridge, 2018; Bowden *et al.*, 2015), which allows for estimation of causal effects in the presence of directional horizontal pleiotropy, but assumes that the SNP strength is independent of the direct SNP effect on the outcome (InSIDE assumption); and b) weighted median MR (Bowden *et al.*, 2016), which is based on the median Wald estimate and allows for consistent estimation even when up to 50% of the information comes from invalid, pleiotropic SNPs. Additional assumptions for the two-sample setting of the present study include that sample should represent the same underlying population (in the present study, a European ancestry population) with minimal sample overlap between studies. We additionally test the reliability of our estimates by replicating the recently published paper’s results (Baranova *et al.*, 2023). We rerun the ADHD to T2D analysis based on the same method and dataset used in Baranova’s study, which used 26 SNPs of ADHD and the updated T2D GWAS data (Mahajan *et al.*, 2022).

In this study, the combined mediation effects were quantified via multivariable Mendelian Randomization (MVMR), in which we firstly obtained the direct effect of ADHD and the indirect effect of mediators’ combination then be estimated by subtracting the direct effect from the total effect. The proportion mediated is calculated by dividing the indirect effect by the total effect. In this case, the indirect effect and the proportion mediated are the function of total effect and direct effect, therefore, the standard error (SE) and confidence intervals (CI) can be calculated through the bootstrap method or the delta method. Functions are listed below:

$$Indirect effect=Total effect-Direct effect$$

$$Proportion mediated={Indirect effect\times100\%}/{Total effect}$$

The bootstrap is a simulation method for computing standard errors and distributions of statistics of interest, which employs an estimated data generating process for generating artiﬁcial (bootstrap) samples and computing the (bootstrap) draws of the statistic. As the bootstrap method is less restrictive than the delta method, for example, bootstrapping does not require normality, bootstrapping is a more accurate method of calculating confidence intervals or standard errors for transformations of parameters (Mandel, 2013). The general process of bootstrap is below:


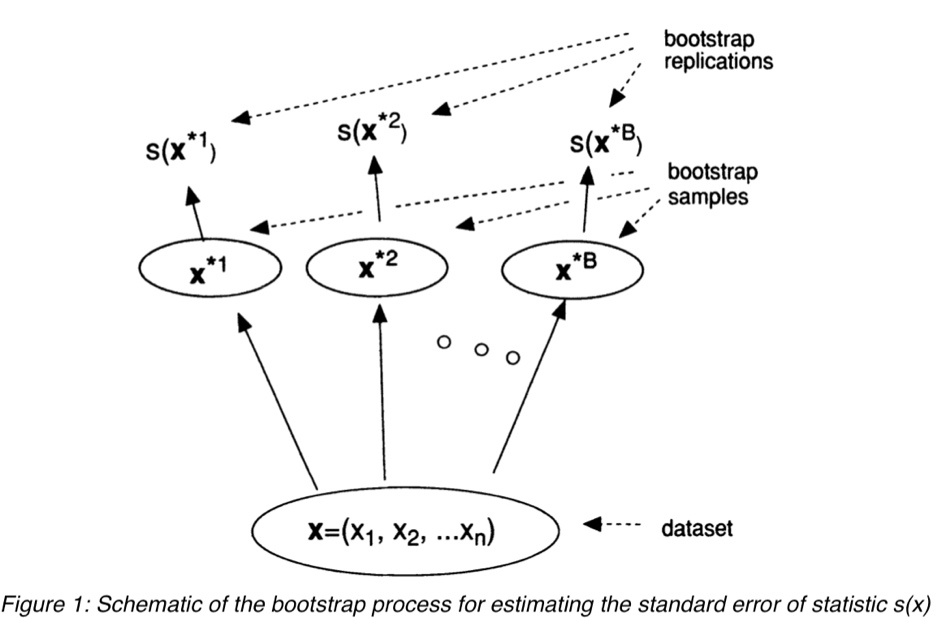


Figure 1. Schematics of the bootstrap process for estimating the standard error (Beran, 2008)

For the bootstrap method CI calculation, we firstly built models of total effect (univariable MR) and direct effect (MVMR). Then extracting the corresponding coefficients of total effect and direct effect to formulate the function of indirect effect and proportion mediated. The last step is to use the nonparametric bootstrapping based on 10,000 bootstrap sample estimates to generate the CI of each function (Canty, 2002). The bootstrapping was done via the “boot” R package.

The delta-method involves calculating the expansion up to the first order of a Taylor series approximation of a function (Herberg and Bristol, 1962) to derive the empirical value and the SE of an estimator. For example, an approximation to the covariance matrix of g(X) is given by:

$$Cov\left( g\left( X \right) \right)=g^{'}\left( \mu\right)Cov\left( X \right)\left[ g^{'}\left( \mu\right) \right]T$$

Following the recommendation in a recent review on MR methods for mediation (Carter et al., 2021), we have additionally used the delta method to validate the CIs. The delta method estimation of SE includes three steps. The first step is to model the total effect (univariable MR) and direct effect (MVMR). The second step is to extract the coefficients and the variances of each estimate, which is used to construct the covariance matrix. At last, we calculated the SE of indirect effect and proportion mediated using the “deltamethod” function of “msm” R package (Jackson, 2011).

**ESM Figure 1.** Estimates of the proportion mediated by each individual mediator using the difference in coefficient method


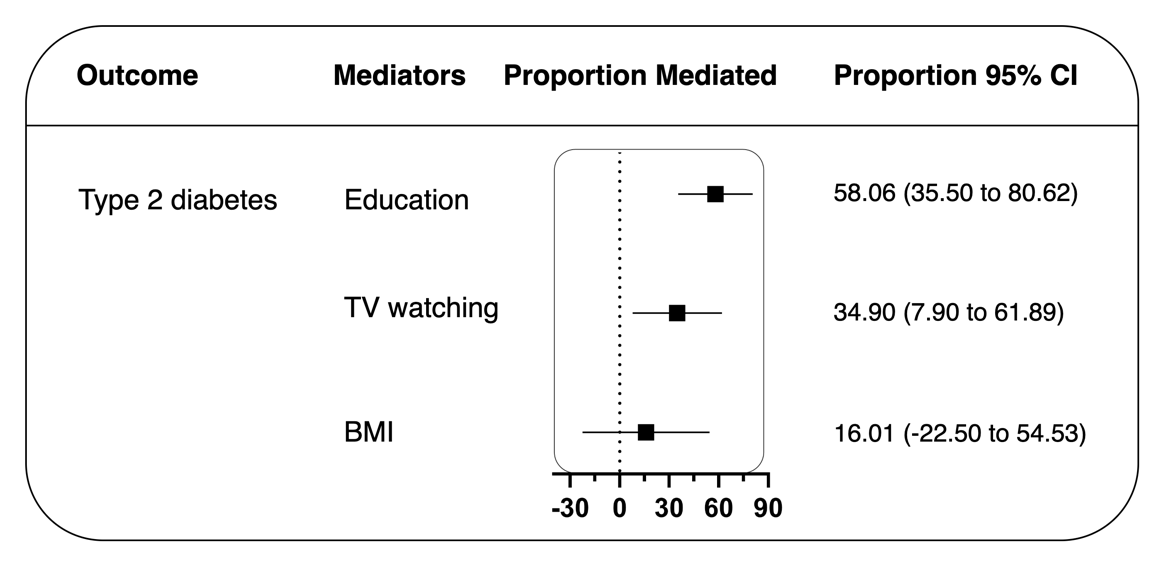


| **ESM Figure 2**. Reverse Mendelian randomization scatterplots (mediators to ADHD) | | |
| --- | --- | --- |
| **A.** BMI > ADHD | **B.** SBP > ADHD | **C.** DBP > ADHD |
| 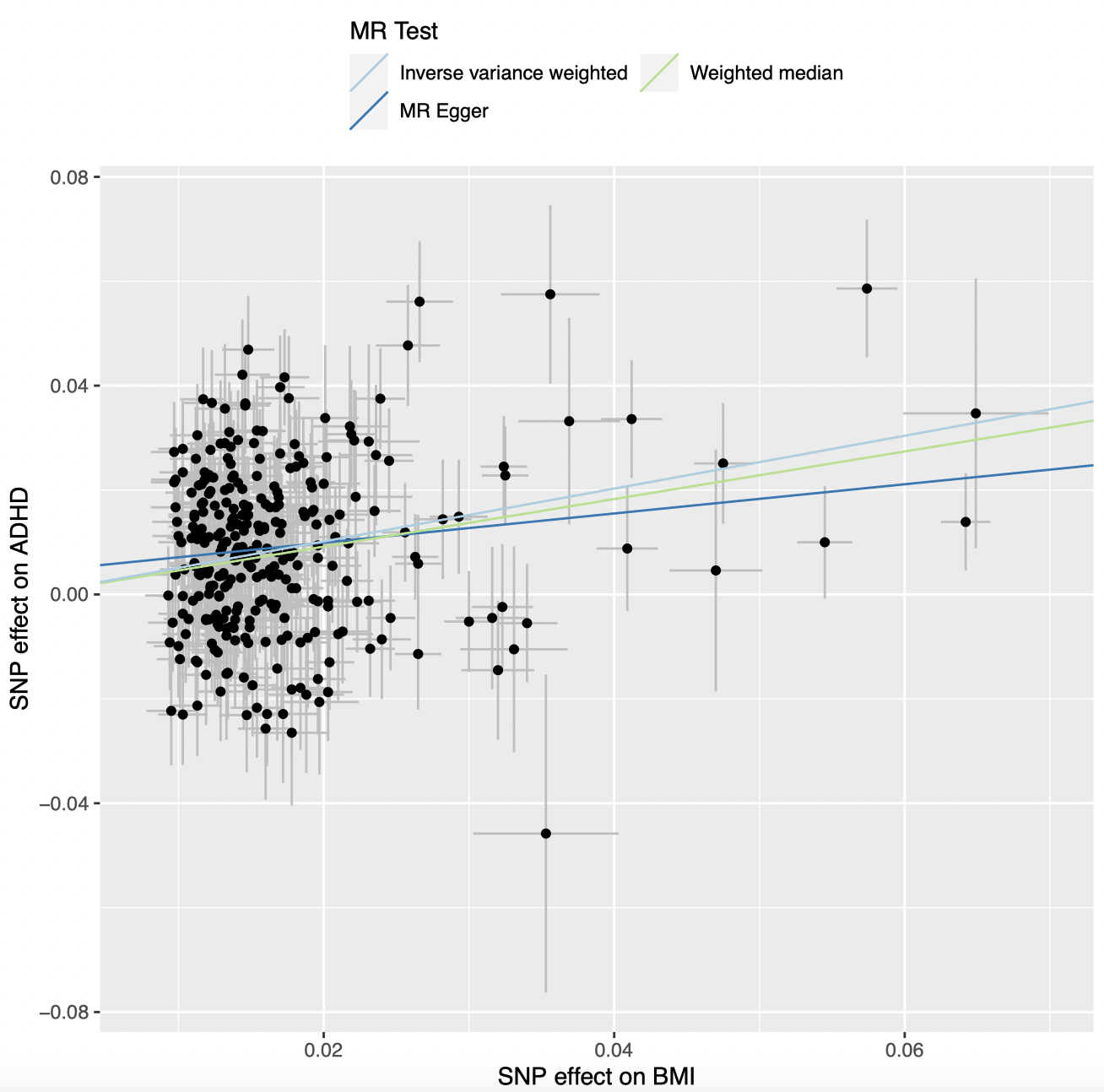 | 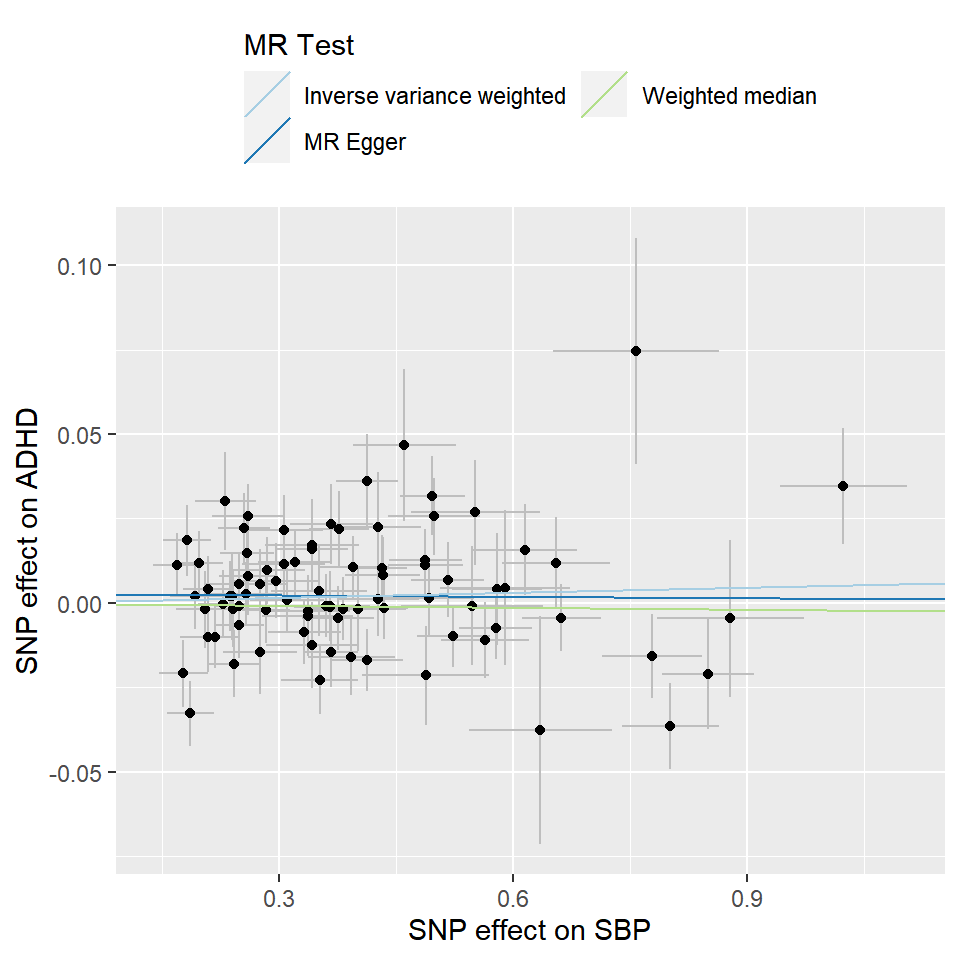 | 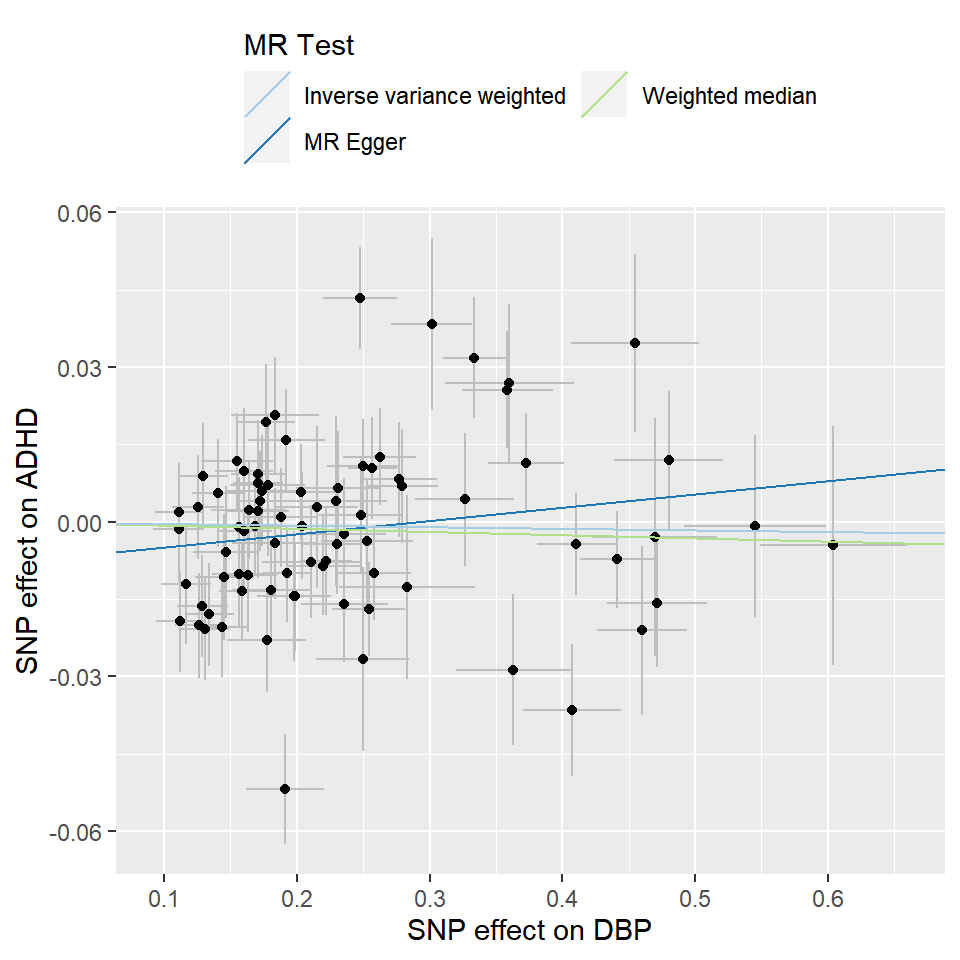 |
| **D.** TV watching > ADHD | **E.** Smoking > ADHD | **F.** Childhood BMI > ADHD |
| 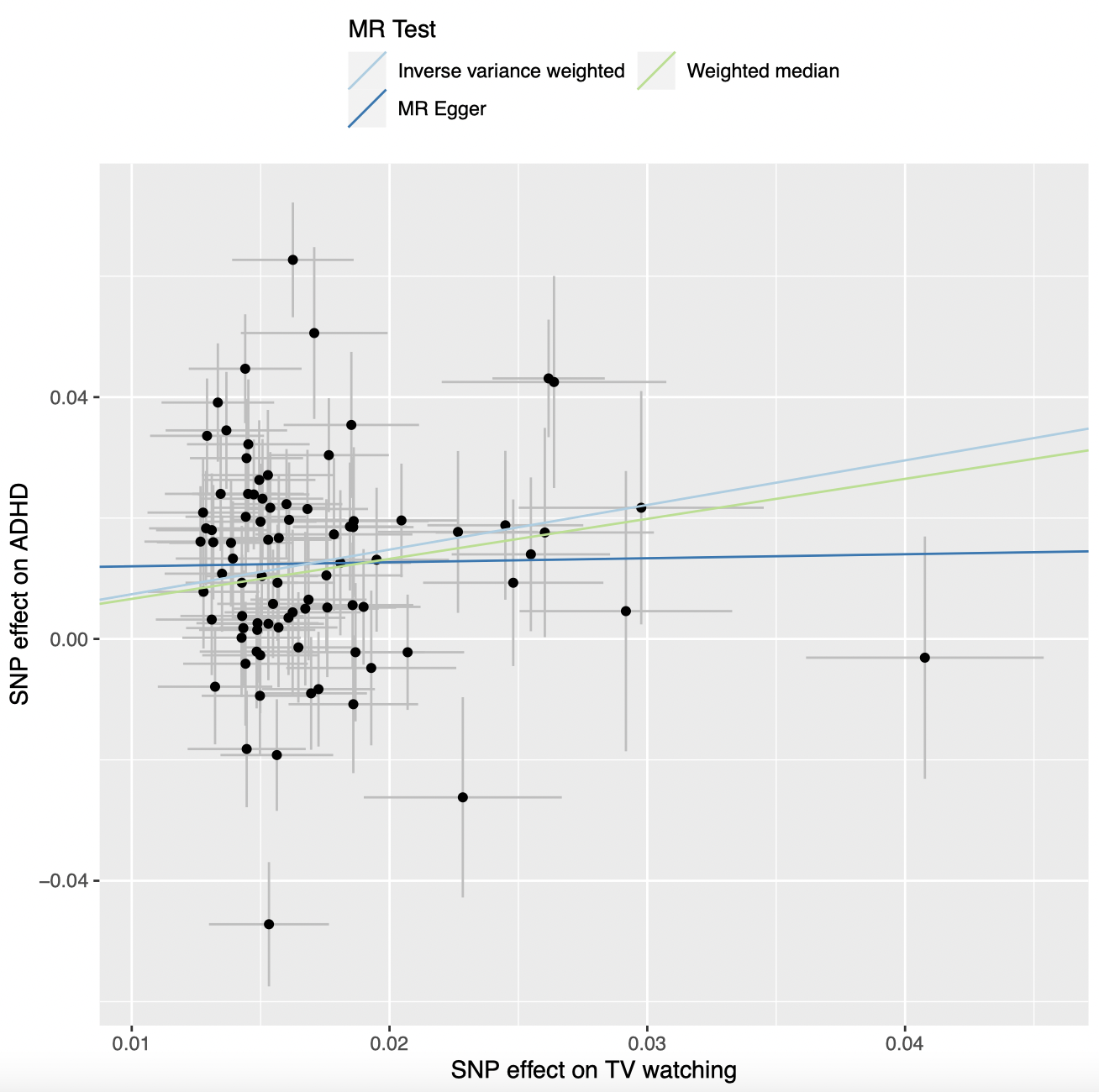 | 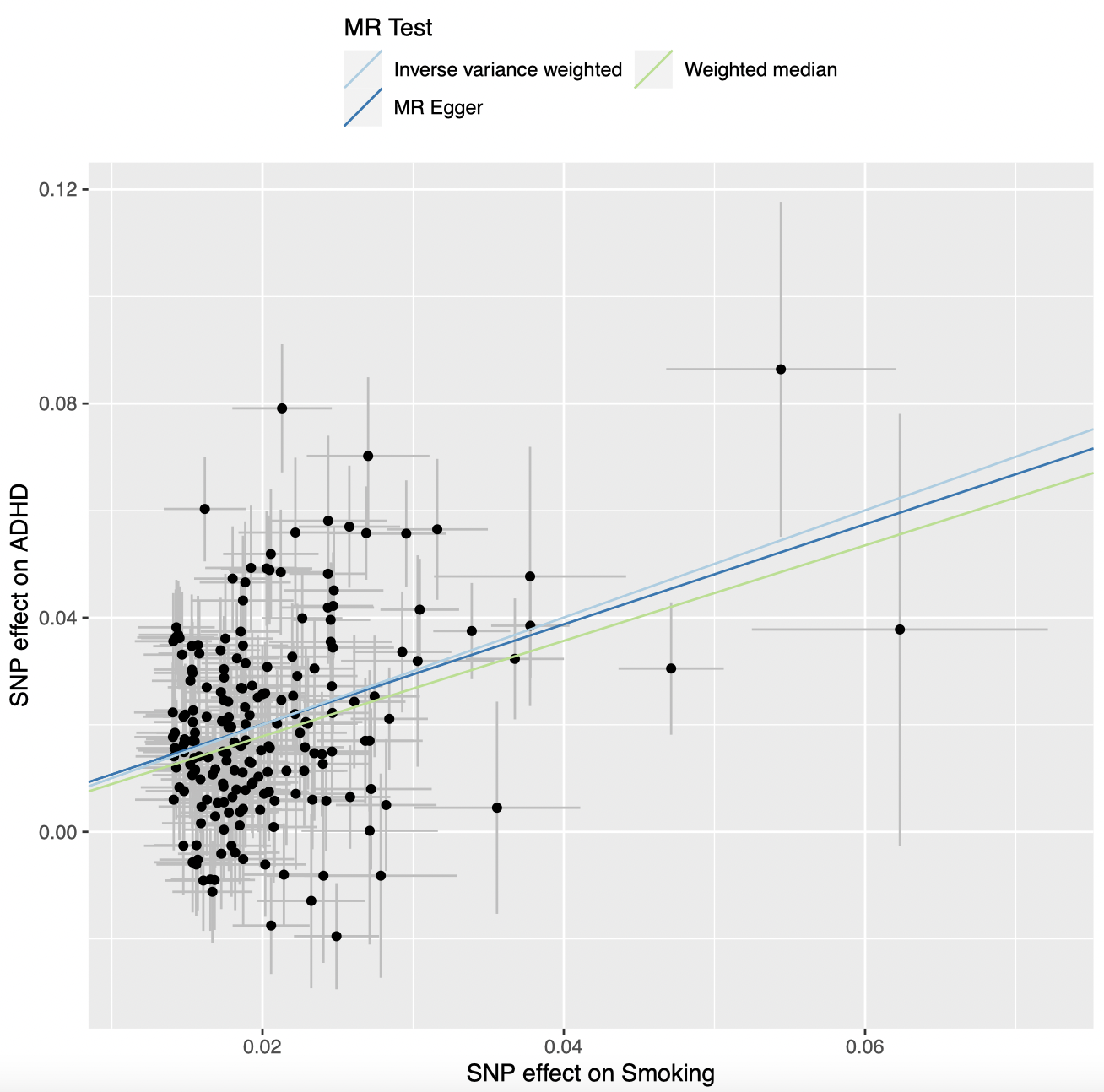 | 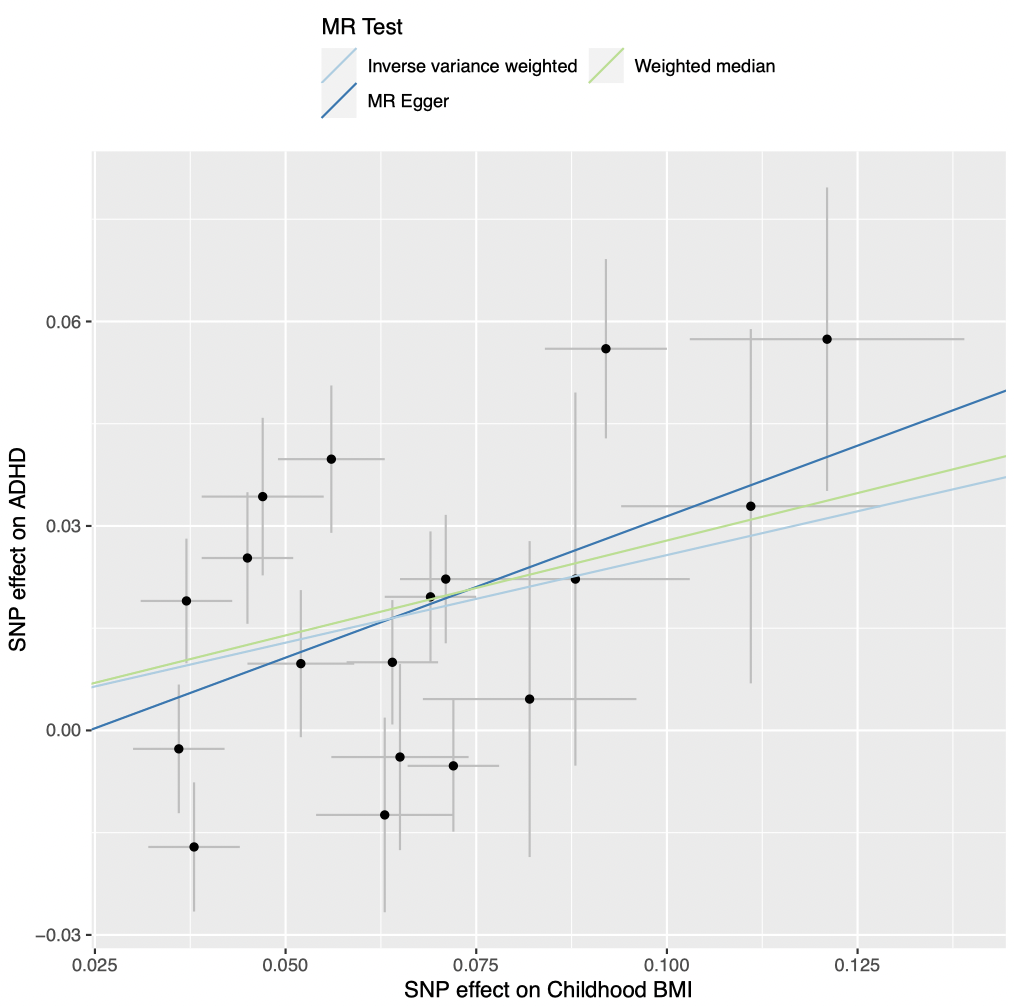 |
| **G.** Childhood obesity > ADHD | **H.** Educational attainment > ADHD | **I.** CRP > ADHD |
| 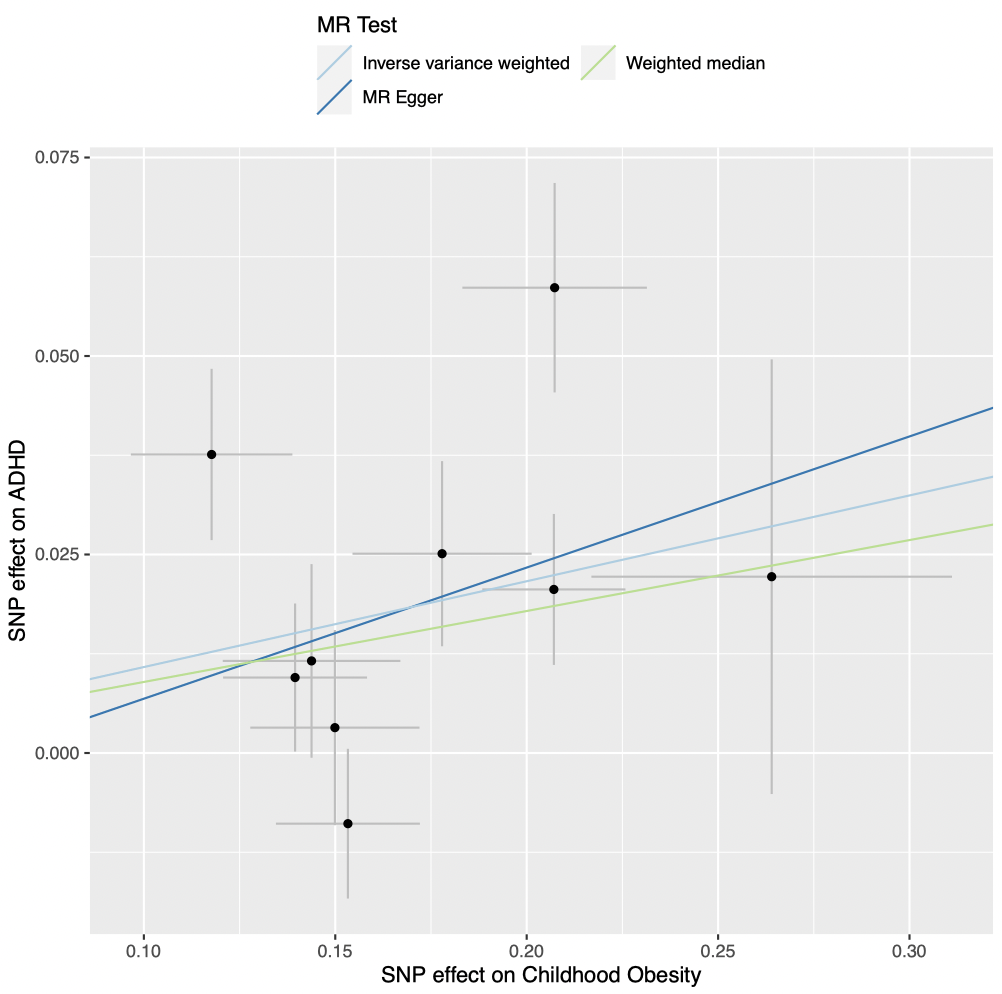 | 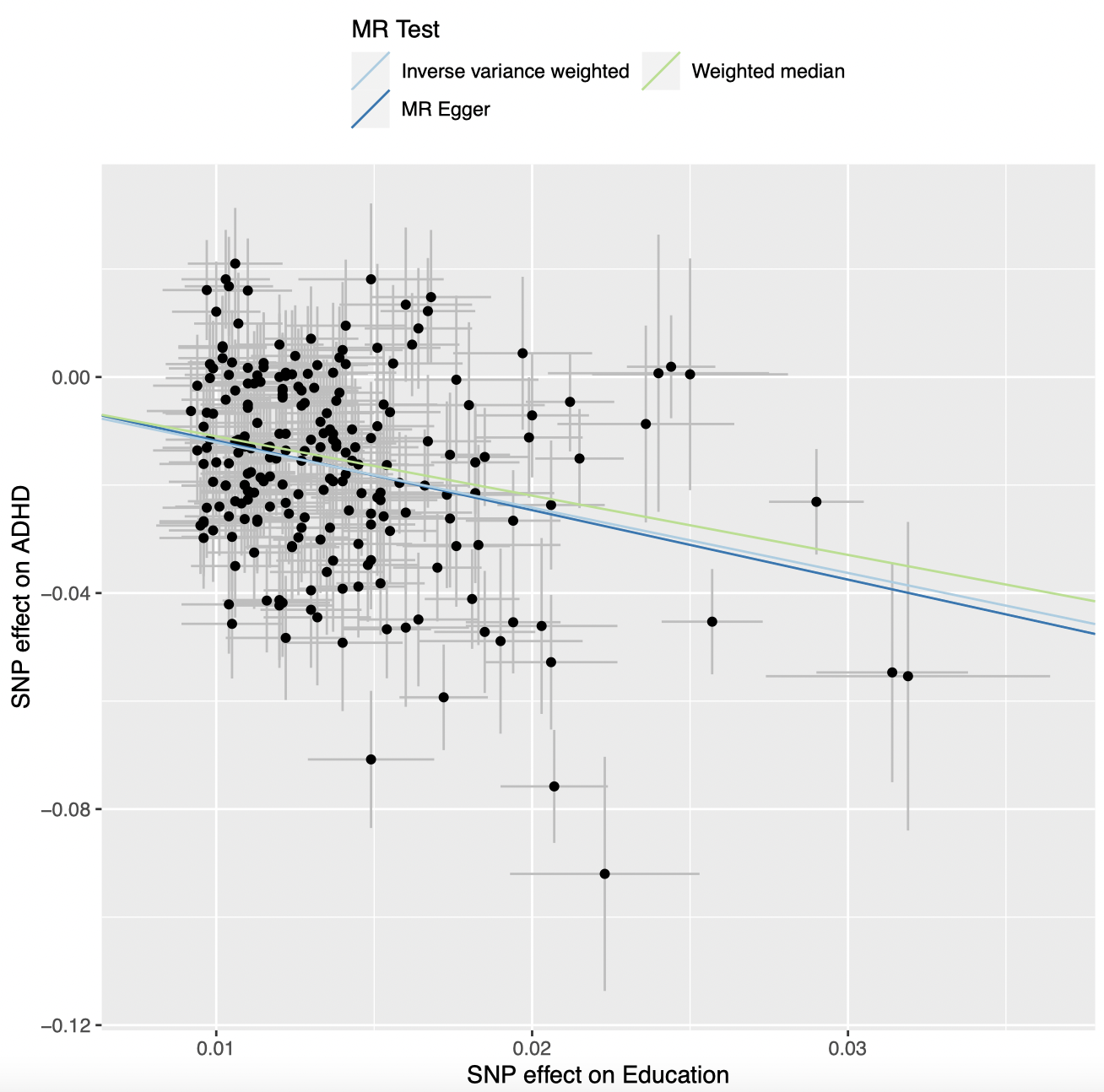 | 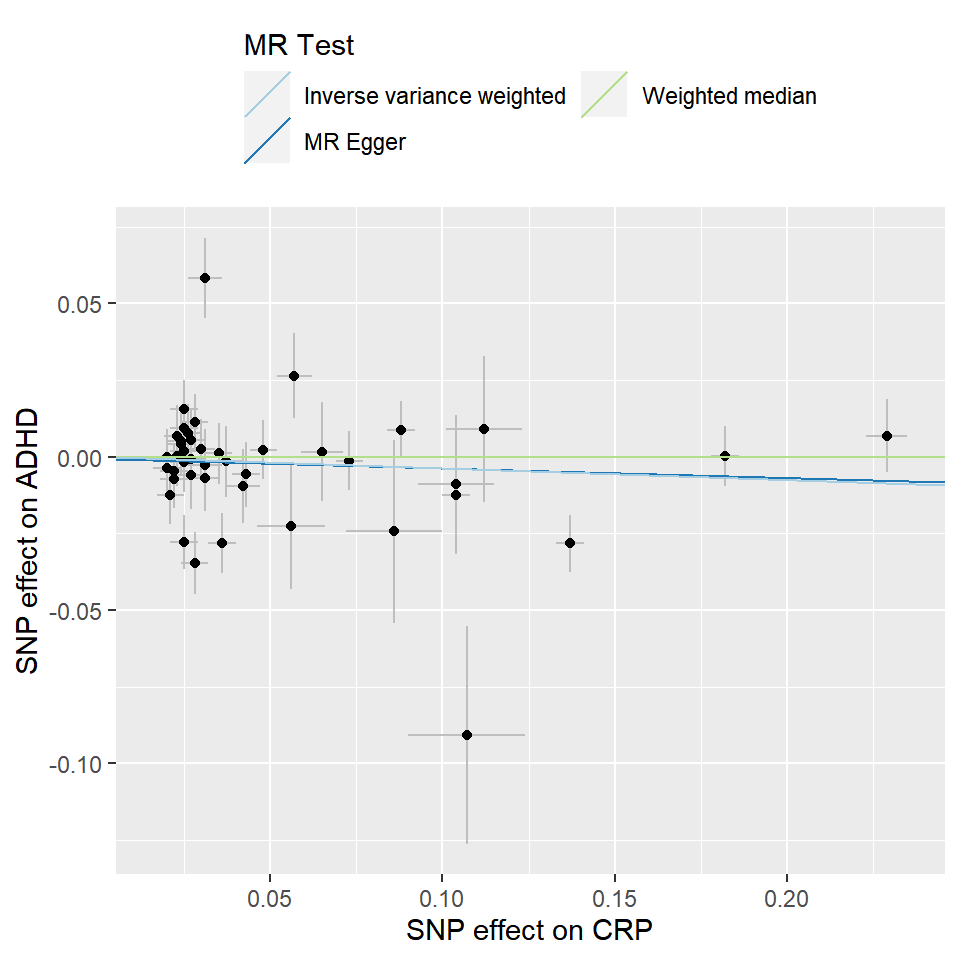 |
| Mendelian randomization scatterplots. Y-axes represent SNP effects on ADHD. X-axes represent SNP effects on (**A**) BMI, (**B**) Systolic blood pressure, (**C**) Diastolic blood pressure, (**D**) TV watching, (**E**) Smoking (ever vs never), (**F**) Childhood BMI, (**G**) Childhood obesity, (**H**) Educational attainment, and (**I**) CRO. The lines represent pooled estimates of causal effects from inverse variance weighted analysis (lightblue), MR-Egger regression (darkblue), and weighted median analysis (yellow). | | |

**ESM References**

**Baranova A, Chandhoke V, Cao H and Zhang F**. (2023) Shared genetics and bidirectional causal relationships between type 2 diabetes and attention-deficit/hyperactivity disorder. *General Psychiatry* **36**: e100996.

**Beran R**. (2008) An Introduction to the Bootstrap. In: Morris CN and Tibshirani R (eds) *The Science of Bradley Efron: Selected Papers.* New York, NY: Springer New York, 288-294.

**Bowden J, Davey Smith G and Burgess S**. (2015) Mendelian randomization with invalid instruments: effect estimation and bias detection through Egger regression. *Int J Epidemiol* **44**: 512-525.

**Bowden J, Davey Smith G, Haycock PC and Burgess S**. (2016) Consistent Estimation in Mendelian Randomization with Some Invalid Instruments Using a Weighted Median Estimator. *Genet Epidemiol* **40**: 304-314.

**Canty A**. (2002) Resampling methods in R: The boot package. *R News* **2**: 2-7.

**Carter AR, Sanderson E, Hammerton G, Richmond RC, Davey Smith G, Heron J, Taylor AE, Davies NM and Howe LD**. (2021) Mendelian randomisation for mediation analysis: current methods and challenges for implementation. *European Journal of Epidemiology* **36**: 465-478.

**Jackson C**. (2011) Multi-State Models for Panel Data: The msm Package for R. *Journal of Statistical Software* **38**: 1 - 28.

**Lawlor DA, Harbord RM, Sterne JA, Timpson N and Davey Smith G**. (2008) Mendelian randomization: using genes as instruments for making causal inferences in epidemiology. *Stat Med* **27**: 1133-1163.

**Mahajan A, Spracklen CN, Zhang W, Ng MCY, Petty LE, Kitajima H, Yu GZ, Rüeger S, Speidel L, Kim YJ, Horikoshi M, Mercader JM, Taliun D, Moon S, Kwak SH, Robertson NR, Rayner NW, Loh M, Kim BJ, Chiou J, Miguel-Escalada I, Della Briotta Parolo P, Lin K, Bragg F, Preuss MH, Takeuchi F, Nano J, Guo X, Lamri A, Nakatochi M, Scott RA, Lee JJ, Huerta-Chagoya A, Graff M, Chai JF, Parra EJ, Yao J, Bielak LF, Tabara Y, Hai Y, Steinthorsdottir V, Cook JP, Kals M, Grarup N, Schmidt EM, Pan I, Sofer T, Wuttke M, Sarnowski C, Gieger C, Nousome D, Trompet S, Long J, Sun M, Tong L, Chen WM, Ahmad M, Noordam R, Lim VJY, Tam CHT, Joo YY, Chen CH, Raffield LM, Lecoeur C, Prins BP, Nicolas A, Yanek LR, Chen G, Jensen RA, Tajuddin S, Kabagambe EK, An P, Xiang AH, Choi HS, Cade BE, Tan J, Flanagan J, Abaitua F, Adair LS, Adeyemo A, Aguilar-Salinas CA, Akiyama M, Anand SS, Bertoni A, Bian Z, Bork-Jensen J, Brandslund I, Brody JA, Brummett CM, Buchanan TA, Canouil M, Chan JCN, Chang LC, Chee ML, Chen J, Chen SH, Chen YT, Chen Z, Chuang LM, Cushman M, Das SK, de Silva HJ, Dedoussis G, Dimitrov L, Doumatey AP, Du S, Duan Q, Eckardt KU, Emery LS, Evans DS, Evans MK, Fischer K, Floyd JS, Ford I, Fornage M, Franco OH, Frayling TM, Freedman BI, Fuchsberger C, Genter P, Gerstein HC, Giedraitis V, González-Villalpando C, González-Villalpando ME, Goodarzi MO, Gordon-Larsen P, Gorkin D, Gross M, Guo Y, Hackinger S, Han S, Hattersley AT, Herder C, Howard AG, Hsueh W, Huang M, Huang W, Hung YJ, Hwang MY, Hwu CM, Ichihara S, Ikram MA, Ingelsson M, Islam MT, Isono M, Jang HM, Jasmine F, Jiang G, Jonas JB, Jørgensen ME, Jørgensen T, Kamatani Y, Kandeel FR, Kasturiratne A, Katsuya T, Kaur V, Kawaguchi T, Keaton JM, Kho AN, Khor CC, Kibriya MG, Kim DH, Kohara K, Kriebel J, Kronenberg F, Kuusisto J, Läll K, Lange LA, Lee MS, Lee NR, Leong A, Li L, Li Y, Li-Gao R, Ligthart S, Lindgren CM, Linneberg A, Liu CT, Liu J, Locke AE, Louie T, Luan J, Luk AO, Luo X, Lv J, Lyssenko V, Mamakou V, Mani KR, Meitinger T, Metspalu A, Morris AD, Nadkarni GN, Nadler JL, Nalls MA, Nayak U, Nongmaithem SS, Ntalla I, Okada Y, Orozco L, Patel SR, Pereira MA, Peters A, Pirie FJ, Porneala B, Prasad G, Preissl S, Rasmussen-Torvik LJ, Reiner AP, Roden M, Rohde R, Roll K, Sabanayagam C, Sander M, Sandow K, Sattar N, Schönherr S, Schurmann C, Shahriar M, Shi J, Shin DM, Shriner D, Smith JA, So WY, Stančáková A, Stilp AM, Strauch K, Suzuki K, Takahashi A, Taylor KD, Thorand B, Thorleifsson G, Thorsteinsdottir U, Tomlinson B, Torres JM, Tsai FJ, Tuomilehto J, Tusie-Luna T, Udler MS, Valladares-Salgado A, van Dam RM, van Klinken JB, Varma R, Vujkovic M, Wacher-Rodarte N, Wheeler E, Whitsel EA, Wickremasinghe AR, van Dijk KW, Witte DR, Yajnik CS, Yamamoto K, Yamauchi T, Yengo L, Yoon K, Yu C, Yuan JM, Yusuf S, Zhang L, Zheng W, Raffel LJ, Igase M, Ipp E, Redline S, Cho YS, Lind L, Province MA, Hanis CL, Peyser PA, Ingelsson E, Zonderman AB, Psaty BM, Wang YX, Rotimi CN, Becker DM, Matsuda F, Liu Y, Zeggini E, Yokota M, Rich SS, Kooperberg C, Pankow JS, Engert JC, Chen YI, Froguel P, Wilson JG, Sheu WHH, Kardia SLR, Wu JY, Hayes MG, Ma RCW, Wong TY, Groop L, Mook-Kanamori DO, Chandak GR, Collins FS, Bharadwaj D, Paré G, Sale MM, Ahsan H, Motala AA, Shu XO, Park KS, Jukema JW, Cruz M, McKean-Cowdin R, Grallert H, Cheng CY, Bottinger EP, Dehghan A, Tai ES, Dupuis J, Kato N, Laakso M, Köttgen A, Koh WP, Palmer CNA, Liu S, Abecasis G, Kooner JS, Loos RJF, North KE, Haiman CA, Florez JC, Saleheen D, Hansen T, Pedersen O, Mägi R, Langenberg C, Wareham NJ, Maeda S, Kadowaki T, Lee J, Millwood IY, Walters RG, Stefansson K, Myers SR, Ferrer J, Gaulton KJ, Meigs JB, Mohlke KL, Gloyn AL, Bowden DW, Below JE, Chambers JC, Sim X, Boehnke M, Rotter JI, McCarthy MI and Morris AP**. (2022) Multi-ancestry genetic study of type 2 diabetes highlights the power of diverse populations for discovery and translation. *Nat Genet* **54**: 560-572.

**Mandel M**. (2013) Simulation-Based Confidence Intervals for Functions With Complicated Derivatives. *The American Statistician* **67**.

**Schmidt AF and Dudbridge F**. (2018) Mendelian randomization with Egger pleiotropy correction and weakly informative Bayesian priors. *Int J Epidemiol* **47**: 1217-1228.
